# Thermoplastic Processing of PLA/Cellulose Nanomaterials Composites

**DOI:** 10.3390/polym10121363

**Published:** 2018-12-09

**Authors:** T. C. Mokhena, J. S. Sefadi, E. R. Sadiku, M. J. John, M. J. Mochane, A. Mtibe

**Affiliations:** 1 Department of Chemistry, Nelson Mandela University, Port Elizabeth 6031, South Africa; MJohn@csir.co.za; 2 CSIR Materials Science and Manufacturing, Polymers and Composites, Port Elizabeth 6000, South Africa; amtibe@csir.co.za; 3 School of Natural and Applied Sciences, Sol Plaatje University, Kimberley 8301, South Africa; 4 Institute for NanoEngineering Research (INER), Department of Chemical, Metallurgical and Materials Engineering, Faculty of Engineering and the Built Environment, Tshwane University of Technology, Pretoria 0001, South Africa; SadikuR@tut.ac.za; 5 Department of Life Sciences, Central University of Technology Free State, Private Bag X20539, Bloemfontein 9301, South Africa; mochane.jonas@gmail.com

**Keywords:** polylactic acid (PLA), cellulose nanomaterials, composites, functionalization, properties

## Abstract

Over the past decades, research has escalated on the use of polylactic acid (PLA) as a replacement for petroleum-based polymers. This is due to its valuable properties, such as renewability, biodegradability, biocompatibility and good thermomechanical properties. Despite possessing good mechanical properties comparable to conventional petroleum-based polymers, PLA suffers from some shortcomings such as low thermal resistance, heat distortion temperature and rate of crystallization, thus different fillers have been used to overcome these limitations. In the framework of environmentally friendly processes and products, there has been growing interest on the use of cellulose nanomaterials viz. cellulose nanocrystals (CNC) and nanofibers (CNF) as natural fillers for PLA towards advanced applications other than short-term packaging and biomedical. Cellulosic nanomaterials are renewable in nature, biodegradable, eco-friendly and they possess high strength and stiffness. In the case of eco-friendly processes, various conventional processing techniques, such as melt extrusion, melt-spinning, and compression molding, have been used to produce PLA composites. This review addresses the critical factors in the manufacturing of PLA-cellulosic nanomaterials by using conventional techniques and recent advances needed to promote and improve the dispersion of the cellulosic nanomaterials. Different aspects, including morphology, mechanical behavior and thermal properties, as well as comparisons of CNC- and CNF-reinforced PLA, are also discussed.

## 1. Introduction

Over the past decades, there has been a tremendous interest in the utilization of nano-sized particles, such as layered silicates, carbon nanomaterials and metals, as suitable reinforcements of different polymeric materials towards various advanced applications [1,2,3]. The presence of these nano-sized particles significantly improves the resulting properties, i.e., thermal, mechanical and barrier properties, at fairly low contents (<10 wt %) [1,2,4]. Due to the growing environmental concerns, there has been considerable effort dedicated to the use of eco-friendly particles. Cellulose nanomaterials (CNMs), due to their abundant availability, renewability, biodegradability, high strength and stiffness, are considered suitable replacements of abovementioned nano-sized particles [3,5,6].

CNMs include all nano-sized cellulose-based particles having various shapes, sizes and surface chemistries and properties. CNMs can be extracted from different sources, namely woody/non-woody, tunicates and algae, or generated by bacteria. Since the biosynthesis of these sources differs, the resulting cellulose nanomaterials have different degrees of crystallinity, aspect ratios and morphologies [7]. CNMs can be isolated either through mechanical treatment or acid hydrolysis, or the combination of these processes, which will result in different morphologies and surface chemistries [4]. Nonetheless, CNMs exhibit high aspect ratio, Young’s modulus of ~114 GPa, and tensile strength of ~6000 MPa that are comparable to the commonly used inorganic fillers, which are, however, not biodegradable and renewable [4,7,8]. Thus, CNMs can be exploited as reinforcement of different polymeric materials towards various applications.

Over the past couple of years, the reinforcement of biopolymers (e.g., polylactic acid (PLA), poly(caprolactone) (PCL), poly(butylene adipate terephthalate) (PBAT), polyhydroxy alkanoates (PHAs), etc.) has received tremendous interest as alternative substitution for petroleum-based polymers towards a wide variety of applications because of the stringent environmental legislations [4,9,10,11,12] in place in several countries. This is owing to their unique features, such as eco-friendliness, biocompatibility and ease of processability. Moreover, biopolymers degrade into harmless constituent elements when disposed-off after their intended usage. Among them, PLA merits special interest due to its outstanding properties when compared to petroleum-based polymers (e.g., polypropylene and poly (ethylene terephthalate)) and easy processability by film processing, injection molding and blow molding techniques [9,10,13].

CNM-reinforced PLA qualify to be termed “green nanocomposites”, which is of significance with regard to environmental issues since both PLA and CNMs are biodegradable and renewable [14,15]. Furthermore, the presence of the CNMs can result in improve mechanical, thermal and thermo-mechanical properties of PLA to afford their application in various fields, e.g., packaging and biomedical. It is recognized that CNMs are inherently hydrophilic, which in turn, leads to their inhomogeneous dispersion in hydrophobic PLA, thus adversely affecting the resulting properties. Research has escalated, however, in the functionalization of CNMs to overcome poor dispersion and/or adhesion with PLA, and this aspect is discussed in this review. Moreover, this review gives an overview on the preparation of PLA/CNMs nanocomposites by using thermoplastic processing technique and their properties by highlighting the differences among cellulose nanomaterials, CNFs and CNCs.

### 1.1. Polylactic Acid (PLA)

PLA is an aliphatic polyester biopolymer that can be derived from renewable sources, such as corn, potato, molasses, tapioca, cane sugar, and rice [16]. From these renewable sources, lactic acid is basically produced by a fermentation process and used as monomer to synthesized PLA through different polymerization routes, viz. ring opening polymerization (ROP), polycondensation and other direct methods (e.g., azeotropic dehydration and enzymatic polymerization) [4]. PLA is available in the market with different molecular weights. ROP affords the production of high molecular weight PLA when compared to polycondensation technique method of production. Thus, the two main PLA industrial producers, i.e., NatureWorks LLC and Corbion, mostly use ROP. Other companies, such as Cargill Dow Polymer LCC, Shimadzu Corp, Mitsui Chemicals, Musashino Co., also produce PLA towards various commercial applications (e.g., packaging, textiles, pharmaceutical products and biomedical devices) [4,11,13].

**Figure 1:**
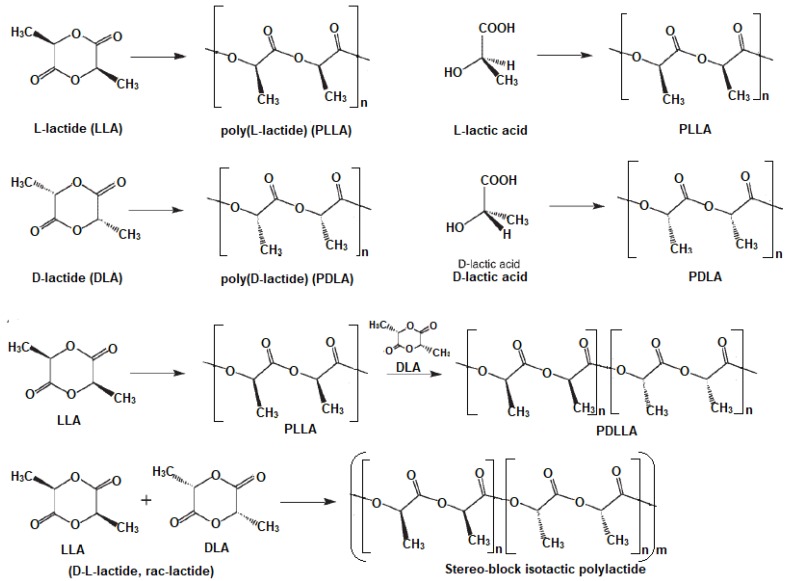
Synthesis and chemical structures of lactide stereoisomers and copolymers.

PLA has at least three stereoisomers, namely poly(l-lactide) (PLLA), Poly(d-lactide) and poly(dl-lactide) (PDLLA), which result from the presence of two chiral carbon centers (Figure 1) [4,17]. Therefore, it is possible to produce isotactic l-PLA and d-PLA polymers as well as dl-PLA composed of syndiotactically alternating d,l-copolymer or a stereo-block isotactic copolymer consist of l- and d-units (Figure 1) [16,17]. In this review, to avoid any confusion, PLA is used to describe all PLA-based polymers. The properties of PLA are influenced by several factors, such as source, the component isomers, the processing routes and molecular weights. It is mainly affected by stereochemistry and thermal history, which also influence its crystallinity and, therefore, resulting properties. For instance, when PLLA content is higher than 90%, it tends to be highly crystalline, while melting temperature (*T*_m_) and glass transition temperature (*T*_g_) decrease on decreasing PLLA content. Table 1 presents the main physical properties of PLA-based polymers [4,9,11,13,17].

PLA possesses remarkable properties, which include biocompatibility, UV stability, clarity, and luster. PLA has been exploited in various fields, such as packaging and biomedical with regard to its biodegradability and biocompatibility. There are, however, some limitations that hinder its success, such as slow crystallization, low glass transition and brittleness [4,10]. There has been a lot of effort to modify PLA to overcome these limitations and match the end applications. Several modifications such as blending with other polymers, copolymerizing with functional monomers, aminolysis, and reinforcement with different fillers have been explored as suitable strategies. On the other hand, the use of nanofillers, yielding so-called “nanocomposites materials”, merit special attention due to the capability of these particles to enhance mechanical, and thermo-mechanical properties as well as to provide additional functionalities at fairly low contents, viz. below 10 wt %.

### 1.2. Cellulose Nanomaterials

Cellulose nanomaterials (CNMs) are considered as a suitable solution to replace commonly used and expensive inorganic nanofillers because they are cheap, renewable and biodegradable. Moreover, they possess unique valuable characteristics, such as high specific strength, moduli (100–200 GPa) and specific surface area, as summarized in Table 2. CNMs can be extracted from different natural sources (wood, non-woody and animal materials), by mechanical treatment and acid hydrolysis or combination of the two. They can broadly be grouped into five categories, viz., cellulose nanofibers (CNF), cellulose nanocrystals (CNCs), bacterial cellulose (BC), algal cellulose (AC), and tunicate cellulose depending on the source and extraction method, as discussed below [3,7,18,19,20,21,22,23].

Cellulose nanofibers (CNF): Cellulose nanofibers, also known as either microfibrillated cellulose (MFC) or cellulose microfibrils (CMF), are mostly obtained by mechanical treatment from cellulose-based materials and are recognized by web-like structure that consists of crystalline and amorphous domains [7,34]. CNFs have high tensile modulus, lightweight, surface activity and biocompatibility, hence they have been used as filler in the composites field (packaging and paper industry), a stabilizer in emulsions, a conducting sheet in electronics and a biomaterial in medical application. Typically, extraction processes of CNFs include grinding, high-shear homogenization, high-intensity ultrasonication and cryogenic crushing, followed by purification (e.g., pulping) [34,35]. These high-energy induced mechanical treatments, which are also time-consuming as a result of repeated cycles, hinder their industrial use [35]. Again, prior treatments, such as chemical and/or enzymatic treatment to reduce the energy and time consumption, result in a huge damage on the crystalline phase of the fibrils, thus adversely affecting the mechanical properties. Nonetheless, these drawbacks are overshadowed by the unique valuable properties of CNFs, such as high purity, high specific strength, wettability, abundant availability, biocompatibility and surface-tunable structures, which afford their various biomedical applications, especially in the development of scaffolds for tissue engineering [34].

Bacterial cellulose (BNC): Beside plants being the main source of cellulose, different bacteria can produce cellulose, as initially reported in 1988 by Brown, who identified the growth of unbranched pellicle that has a chemically equivalent structure as plant cellulose [36]. Bacterial cellulose (BNC) possesses unique features, such as high mechanical strength, crystallinity and purity, thus it is mostly utilized as reinforcement material for polymeric networks to anchor onto therapeutic agents or maintain the tensile shape-contour of scaffolds [37]. It is produced extracellularly by gram negative bacterial cultures (*Gluconacetobacter*, *Acetobacter*, *Agrobacterium*, *Achromobacter*, *Aerobacter*, *Sarcina*, *Azobacter*, *Rhizobium*, *Psuedomonas*, *Salmonella* and *Alcaligenes*) in either synthetic or non-synthetic medium, through oxidative fermentation [37]. *Gluconacetobacter* genus is the most efficient BNC producer with high yields in liquid medium [36,37]. The limitations of bacterial cellulose are the production cost (with about 30% overall cost belonging to the cost of fermentation medium), efficient process scale-up, separation methods, purification methods and low yield [36,37,38].

Cellulose nanocrystals (CNC): Cellulose nanocrystals are rod-like crystalline particles isolated from various natural sources via mineral acid hydrolysis. Depending on the extraction conditions and cellulose raw material, nano-sized cellulose crystal of different dimensions (length = 100–1000 nm and diameter = 4–25 nm) and crystallinities (55%–90%) can be obtained. Although sulfuric acid is the most extensively used to afford the isolation of CNCs, other acids, such as phosphostungstic [39], hydrobromic [40], and phosphoric [41] acids and organic acids (maleic [42], formic [43,44] and oxalic acids) are also reported for such purpose.

Algal cellulose (AC): The extraction of cellulose from algae is considered as an environmental bioremediation with regard to their excessive and unwanted blooming, which damages marine ecosystem [34]. For instance, its growth can reduce the transparency of water, hence adversely affect other species that grow deeper in the water due to lack of sunlight. There are three groups of algae species and they are categorized according to their cell wall constituents: (i) Group 1 is composed of native cellulose as the major component of the cell walls, which is usually highly crystalline (e.g., Cladophorale and Siphonocladales orders); (ii) Group 2 consists of mercerized-like cellulose (which is presumably a derivative of native cellulose) and has low degree of crystallinity (e.g., Spongomorpha); and (iii) Group 3 includes heterogeneous algae, in which cellulose is not a major component of the cell walls (e.g., Vaucheria and Spirogyra algae) [45]. The high degree of crystallinity of algae is associated with the presence of thick cellulose microfibrils (width of 10–30 nm), which may differ according to cellulose synthase complexes terminal complexes (TCs). It is recognized that linear TCs produce Iα-rich cellulose, while rosette TCs produce Iβ-dominant cellulose; however, a boundary between Iα-rich and Iβ-dominant may exist in certain algae species [45]. Nevertheless, CNFs and cellulose nanocrystals extracted from either red or brown algae, have been reported in the literature [26,27,34,45,46].

Tunicate cellulose: It is biosynthesized by cellulose synthesizing enzyme complexes in the membrane of epidermis through different mechanisms [47]. It performs different functions in various tunicate families and species, thus different structural diversities from one species to the next [47]. Similar to plants, tunicate cellulose aggregates in the form of microfibrils, are composed of nearly pure cellulose Iβ allomorph. It has a very large aspect ratio, ranging between 1 and 150 (i.e., length = 100 nm to several micrometers and cross-section = 5–10 nm) [48,49]. It also possesses high surface area (150–170 m^2^/g), high crystallinity (95%), high tensile modulus and reactive surface via surface hydroxyl groups, hence it has been used to improve the mechanical properties of composite materials [24,48,49,50].

## 2. Functionalization of Cellulose Nanomaterials

**Figure 2:**
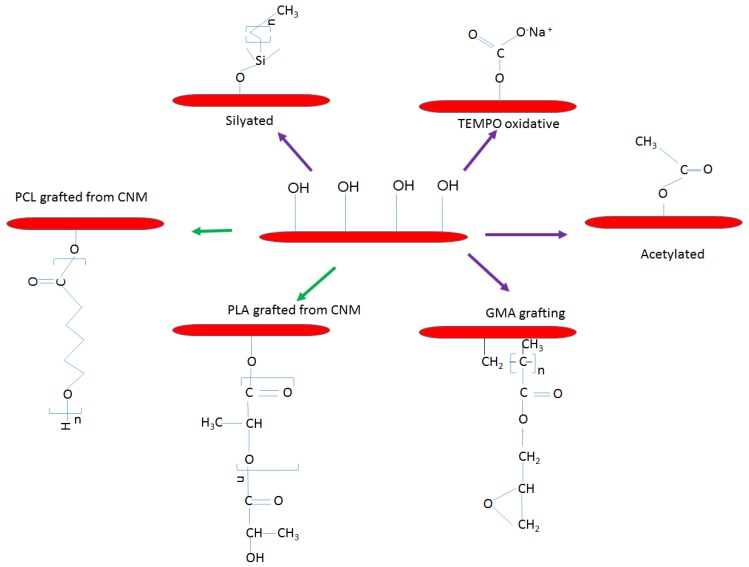
The commonly used chemical modification of CNM in PLA nanocomposites.

It is recognized that the surface properties of CNMs play a major role in the fiber–fiber bonding within cellulose network and the interfacial adhesion between the fiber and the matrix, which in turn, dictates the resulting properties of the nanocomposites. Considerable effort has been dedicated to the optimization of fiber–matrix interface such that exceptional mechanical properties of single CNM can be transferred to the macroscale properties of the bulk nanocomposites, and to obtaining excellent distribution of the CNMs in the continuous polymer matrices. The hydrophilic nature of CNMs fuels their combination with water-soluble polymers, followed by film casting as the preferable preparation route. Surface modification, however, opens the door for the application of CNM as reinforcement of various polymeric materials by using different processing methods, especially classic thermo-processing techniques [51,52]. The surface modification of CNMs can be categorized into three groups: (i) substitution of hydroxyl groups with small molecules (purple arrow); (ii) polymer surface modification by “graft to” strategy with different coupling agents; and (iii) polymer surface modification by “graft onto” strategy with radical polymerization of ring opening polymerization (ROP), atom transfer radical polymerization (ATRP) and single-electron transfer living radical polymerization (green arrows) [53]. In the case of PLA-based nanocomposites, several researchers have reported on the functionalization of CNMs to improve their dispersion and interaction with PLA matrix. The functionalization of CNMs in the PLA system includes: acetylation [54], salinization [55], silylation, glyoxalization, grafting of PCL [56], PLLA [57], PEG or glycidyl methacrylate (GMA) [58,59] and the use of surfactant [60], as summarized in Table 3 and Figure 2. Depending on the type of functionalization, the properties of the resulting composites are improved. Lin et al. [56] reported that the incorporation of PCL-grafted-CNC into PLA matrix improved the tensile strength and elongation. They attributed this to the ability of rigid CNCs to endure higher stress as well as the essential associations of the facile stress transfer to the CNC mediated with grafted PCL chains.

The pre-treatment of the fibers for facilitating their extraction method (refinement step) can also be employed as a strategy to functionalize the ensuing cellulose nanomaterials. For instance, TEMPO oxidation pre-treatment of cellulose fibers introduces carboxylic groups at the surface of the cellulose nanomaterials [61]. In another study, it is reported that carboxymethylation pre-treatment and prior mechanical treatment result in carboxymethylated cellulose nanofibers (CNF) [62].

## 3. Composites Preparation

Firstly, it is important to emphasize the hydrophilic nature of the cellulose nanomaterials due to the presence of hydroxyl groups and this has been one of the difficulties in the promotion of their dispersion in various polymeric materials, regardless of the preparation method. Cellulose nanomaterials have strong attractive forces emanating from the surface hydroxyl groups (–OH) via hydrogen bonding, thereby causing irreversible agglomeration, reorganization and co-crystallization. Chemical modifications are applied to mask these hydroxyl groups to avoid their agglomeration. Since cellulose nanomaterials are obtained in water suspension, solution casting has been a preferable preparation route to avoid irreversible agglomeration during drying and to obtain reasonable dispersion states in aqueous media and some organic solvents [51,54,55,66]. Moreover, this method is often employed to prepare the master-batch when other processing techniques, such as extrusion are employed to prepare PLA/CNM composites [67,68]. To overcome CNM–CNM interactions, considerable efforts have been dedicated to improving the interfacial compatibility by surface functionalization of cellulose nanomaterials or by using different compatibilizers to afford the use of other preparation routes, such as melt compounding, as discussed below [52]. Table 4 summarizes selected studies, based on thermoplastic processing techniques for the production of PLA/cellulose nanomaterials composites.

### 3.1. Compression Molding

**Figure 3:**
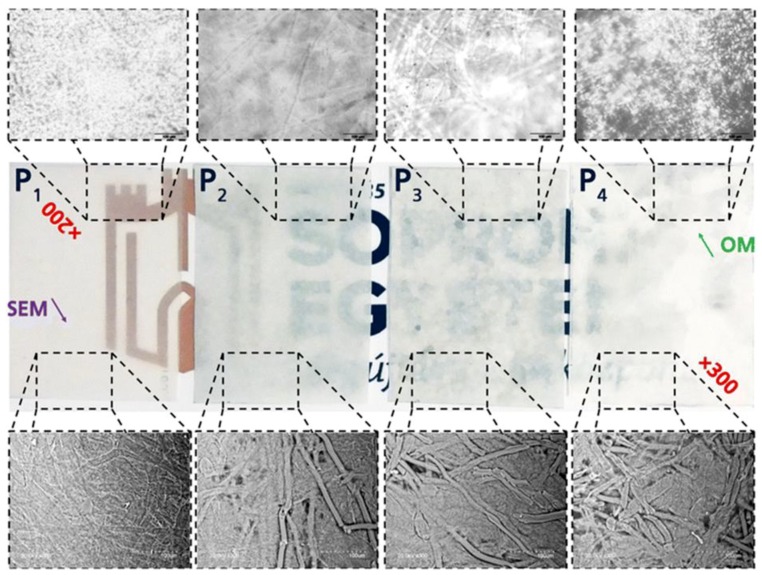
The first row presents optical microscopic images; the second row shows visual appearance; and the third row presents SEM images of film composites—P1, P2, P3 and P4 (Reprinted from [75]; Copyright ©2018, Springer Nature).

Compression molding is often applied to incorporate many cellulose nanomaterials, viz., up to more than 70 wt % [73]. Several studies, based on the preparation of PLA/CNM nanocomposites, have been reported in the literature [72,73,74,75,76]. In most cases, the cellulose nanomaterials are first dried to form a thin paper film, followed by the inclusion of PLA and then compressed at a given pressure and temperature. In other studies, the cellulose nanomaterials are mixed with PLA to obtain homogenous mixtures, followed by the extraction of the solvent and then compression to form sheets [74]. Among these studies, Robles et al. [75] prepared self-bonded composite made of cellulose nanofibers (CNF) and PLA microfibrils, through melt compression molding. The authors mixed 3 wt% CNF suspension with PLA fibrils (PLAF) by using homogenizer, followed by sonication to enhance the interaction between the two. The mixture was then filtered to extract water and hot pressed with hydraulic press at 110 °C, while the pressing cycle was performed as follows: 20 bar for 10 min after closing the press plates, 30 bar for 1 min and then a curing step at a pressure of 150 bar for 5 min. The ratios between CNF and PLAF were 100/0 (P1), 75/25 (P2), 50/50 (P3) and 0/100 (P4). They found that an increase in PLAF content increased the opacity of the nanocomposites films as well as the formation of rough porous material (Figure 3). A nanopaper composed of lactic acid-grafted-CNF is prepared with the aid of compression molding in [76]. The modified nanopaper had smaller density (1.28 gm/cm^3^) when compared to unmodified nanopaper (1.34 gm/cm^3^), which can be attributed to either the separated phases of lactic acid and nanofibers or the porous structure of modified nanopaper, because of trapped air. The latter leads to translucent papers when compared to the transparent unmodified papers. Interestingly, the modified nanopaper absorbed 43% less moisture when compared to the unmodified nanopaper. This can be ascribed to the presence of polymer (oligomer) masking hydroxyl groups (–OH), such that there are few –OH groups that are accessible on the surface of the CNFs. This was confirmed by the amount of water absorbed by the modified nanopaper when soaked in water for 18 h, viz., 35% less than the unmodified samples.

Khakalo, Filpponen and Rojas [72] investigated the effect of casein protein as a potential dispersant in composite films composed of cellulose nanofibers (CNFs) and PLA. The films were hot pressed in a Carver Laboratory press at 80 °C and 1800 Pa for 2 h. Contact adhesion measurements confirmed the effect of surface modification, where ~50% increase in work of adhesion between CNF and PLA was obtained in the presence of casein.

### 3.2. Melt Compounding

Melt compounding serves as the most important polymer processing technique employed today [77]. It serves as an essential integral technique to mix either composites or blends, based on most synthetic polymers, such as PP and LDPE, towards different applications. In this case, the materials pass through the melt compounder one or more times, before the final product is obtained. This technique can be subdivided into two categories: (i) plain melt-mixer; and (ii) single/two-screw extruder, as discussed below.

#### 3.2.1. Melt Mixer

The plain melt mixer is often applied on a laboratory-scale to mix small portions for quick analysis; however, extruders are available in pilot and industrial scales. In the case of melt mixer, the constituents are fed into a bowl that has three independent heating zones and two counter-rotating blades for mixing [78]. The advantages of this technique include small materials required, 40–70 g, exchanging of the blades to afford precise mixing depending on the material, and controllable or programmable temperatures, time and shears (screw speed) to afford nearly finished product. Melt-mixers are often used to establish whether the material used can be implemented in the most widely used industrial melt-processing technique, such as extruders. Raquez et al. [79] studied the effect of surface modification of CNCs and their usage as reinforcements of PLA. Different chemical modifications, based on trialkoxysilanes, such as alkyl, amino and (meth)acryloxy were employed and mixed with PLA by using ThermoHaakeMiniLabRheomex CTW5 mini extruder at 165 °C (100 rpm, 5 min). The resulting unmodified samples were confirmed to be dark by direct visual observation, suggesting that thermal degradation of the CNCs occurred during the processing step. In the case of the chemically modified CNCs, the samples remained colorless, which confirmed that silane treatment can preserve the integrity of CNCs by reducing its thermo-sensitivity and allows its implementation via extrusion. Hong and Kim [78] prepared PLA/CNC composites by melt-mixing (Haake PolyDrive Rheomixer R 600 mixer equipped with a roller blades rotor rotating at 60 rpm), followed by compression molding (Carver hydraulic hot press at 180 °C at a pressure of 1000 Psi for 6 min). Maleic anhydride-grafted PLA was used as compatibilizer to improve the interfacial adhesion between PLA and CNCs. It was found that the optimal processing conditions were: 5 + 10 min, at mixer setting temperature of 190 °C to avoid the PLA and CNC degradation as well as incomplete dispersion of the filler. This was confirmed by performing tensile experiments on the samples. It was found that the highest tensile strength was exhibited by the composite prepared at 190 °C (5 + 10 min) when compared to 180 °C and 200 °C. In the case of the mixing times, 5 + 10 min displayed high tensile strength when compared to 5 + 5 min and 5 + 15 min. The low tensile strength was attributed to the incomplete mixing for the 5 + 5 min, while thermal degradation of PLA was the main reason for the 5 + 15 min processing times. Other biopolymers (e.g., polyhydroxybutyrate (PHB)) can also be utilized as carrier materials to improve the dispersion of the cellulose nanomaterials [80]. Kiziltas et al. [80] prepared a master-batch by mixing PHB and CNF suspension in a bowl mixer, then blended PLA during the second compounding step. It was reported that CNFs were well distributed in the PLA + 5 wt % CNF with the PHB carrier system. The interfacial adhesion between PLA and PHB was also enhanced in the presence of the CNFs, which was associated with the changes in viscosity of the composite.

#### 3.2.2. Extrusion Method

The extruder consists of three processing zones: (i) the feeding zone, where the material is introduced into the barrel; (ii) the kneading zone; and (iii) the heating zone, in which high shears, temperatures and pressures are achieved along with finished product texture, color, density and functional properties. Variables such as screw speed, screw configuration, screw length-to-diameter ratio (*L*/*D*), barrel temperature, feed rates and die shape/size can be programmed or controlled to enable the fabrication of the final product. Bismark and co-workers [77] extruded PLA/CNCs composites by functionalizing the CNCs to promote their dispersion. In this regard, a novel method, based on temperature-induced phase separation (TIPS), were exploited to homogeneously disperse the CNCs in PLA. Freeze-dried CNCs were added to 90 mL of 1.4-dioxane and homogenized at 20,000 rpm, followed by the addition of PLA into the mixture and then mixed overnight at 60 °C under magnetic stirring. The mixture was then poured into a syringe, added drop-wise into a bath of liquid nitrogen to induce phase separation and subsequently freeze-dried to yield porous microsphere composites. The composite films were produced by feeding the desired fraction of CNCs into a twin-screw micro-extruder and kept at a melt temperature of 180 °C and a rotational screw speed of 10 rpm. After the addition of microspheres, the screw speed was increased to 40 rpm for 30 min and then extruded at a screw speed of 20 rpm, followed by pelletizing and compression molding into films. CNCs were chemically modified by using acetic acid (CNC-2), hexanoic acid (CNC-6) and dodecanoic acid (CNC-12). The surface modification of CNCs (i.e., CNC-6 and CNC-12) led to the enhancement in the tensile modulus and tensile strength. Comparison between cellulosic particles sizes as well as chemical modification, viz. 3-aminopropyl triethoxysilane silanized CNFs and dodecanoyl chloride esterified CNCs), was studied by Robles et al. [65]. It was visually observed that the color of the composite samples are similar to that of the pellets obtained from the powdered fibers with different fillers. The salinized samples showed yellowish color, while the esterified CNCs were brownish. It was reported that the esterified CNCs had stronger interaction with the matrix than what was obtained with the salinized CNFs.

Functionalization of the cellulose nanomaterials can affect their location, especially in polymer blends. Elsewhere in the literature, the resultant functionalization of the CNCs by using akyl and PLA chains to control their locations in the PLA/natural rubber (NR) blend [81]. CNC were grafted with *n*-octadecyl isocynate (C18-*g*-CNC) and PLA (PLA-*g*-CNC) chains by in-situ ring opening polymerization of l-lactide. Two methods were employed to prepare the blended nanocomposites, viz. direction extrusion (CNC were lyophilized for 48 h to form a foam, which was pulverized to obtain powder) or solvent casting combined with extrusion. It is reported that a combination of casting and extrusion was necessary to prevent the degradation of the cellulose nanocrystals and to obtain a good dispersion of the fillers. From the authors’ results, an increase in PLA-*g*-CNC concentration led to a progressive reduction of the NR droplet size, while the opposite effect was observed for C18-*g*-CNC. C18-*g*-CNC was located in the NR droplets, whereas PLA-*g*-CNC was located in the PLA phase, thereby increasing the PLA viscosity and, hence, reducing the NR droplets size. Elsewhere, a co-rotating twin-screw extruder with gravimetric feeder for neat PLA and a peristaltic pump for liquid feeding of CNCs and a plasticizer (triethyl citrate) were used [82]. In this regard, atmospheric venting at Zones 2 and 4 and vacuum-venting at the end of the extruder, were employed in order to evacuate the vapor generated during processing. After pelletizing, the films were obtained from compression molding and were cooled either in air (fast cooling) to avoid crystallization or inside the metal plates (slow cooling) to allow crystallization to take place. It was reported that fast cooling resulted in more transparent films when compared to slow cooling, which resulted in haze films. The slow cooled material displayed the presence of spherulites, which are associated to the crystallization of the PLA, hence the haziness of the films.

### 3.3. Melt Spinning

**Figure 4:**
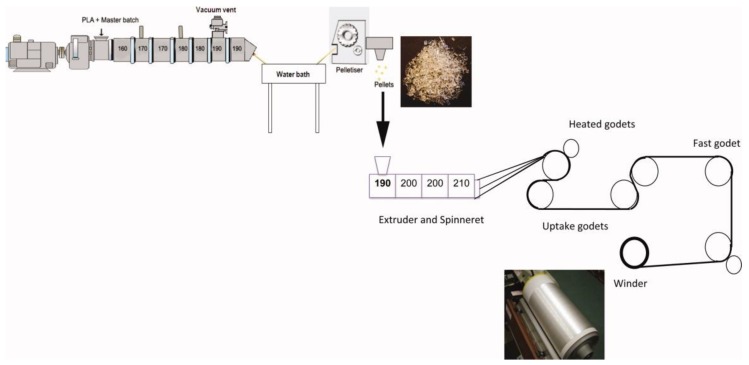
Schematic representation of the melt spinning of PLA and PLA/CNC fibers. Reprinted with permission from [70].

**Figure 5:**
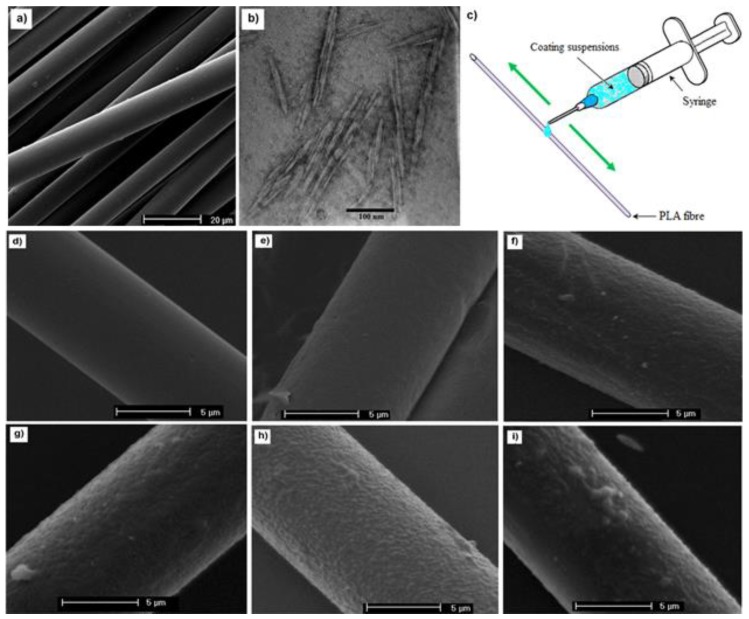
(**a**) Scanning electron microscopy (SEM) image of PLA fibers obtained at 400 m min^−1^; (**b**) Transmission electron microscopy (TEM) of cellulose nanocrystals; (**c**) schematic of the coating procedure employed on the PLA fiber surface; and SEM images of: (**d**) noncoated; (**e**) PLA/PVAc; (**f**) PLA-CNCs-65; (**g**) PLA-CNCs-75; (**h**) PLA-CNCs-85; and (**i**) PLA-CNC-95 fibers. Reprinted from [84] Copyright ©2018 American Chemical Society.

Firstly, it is important to emphasize that melt spinning is often employed with other processing technique(s) to enable the production of nanocomposite. Extrusion is often applied to produce nanocomposites pellets, which are further processed by employing melt filament technique to produce nanocomposite fibers and the morphology of the fibers is found to depend on the content of the CNMs and their functionalization as shown in Figure 4 [70]. Mathew and co-workers [70] prepared microfiber nanocomposites by employing three steps: (i) preparation of master-batch; (ii) extrusion to produce nanocomposite pellets; and (iii) melt-spinning of the pellets to produce the nanocomposite fibers [70]. In their reports, an increase in the fiber diameter with the addition of CNCs from 91 µm to 92.5–94.6 µm was observed due to an increase in the viscosity of the fibers when CNCs were added to the system, thereby leading to relatively low stretching of the fibers. They also observed that the surface roughness increased with increasing content of CNCs because of their aggregation, which resulted in the formation of clusters on the surface of the fibers. It was suggested that surface modification of CNCs or the use of surfactant, can improve their dispersion. Blaker et al. [8] studied the effect of the surface modification of the CNCs on the PLA melt-spun fibers. CNCs were chemically-modified, via organic acid esterification, viz., solvent exchanging into pyridine from water through methanol and then esterified with either hexanoic acid (C-6) or dodecanoic acid (C-12), in the presence of *p*-toluenesulfonyl chloride. As expected, the addition of the CNCs increased the roughness of the fibers and their diameters increased concomitantly with cellulose content due to an increase in melt viscosity. C-6 modified CNC fibers displayed the highest draw ratio (46.7%) when compared to 44.4% for neat PLA and 38.3% for C-12-modified CNCs-based fibers. This was attributed to the improved compatibility observed between PLA and C-6 modified CNCs. PLA-grafted maleic anhydride (PLA-g-MA) as compatibilizer for CNCs/microcrystalline cellulose (MCC) and PLA in the fabrication of PLA composite fibers, was recently reported by Aouat et al. [83]. It was reported that small aggregations of CNCs were visible on the PLA/PLA-g-MA/CNC (1 wt %) surface, which became more pronounced in PLA/PLA-*g*-MA/MCC (1 wt %). This was attributed to the CNCs having higher aspect ratio and smaller size, which led to a stronger interaction with PLA than with MCC. It was also found that at a filler content of 1 wt % and a draw ratio (DR) of 1.5, the average diameter of the compatibilized PLA/CNC was 60 µm, while that of compatibilized PLA/MCC was 65 µm, which was related to the size of the MCC. In general, the diameter of the fibers increased concomitantly with the cellulose content, which can be related by a decrease in the spinning rate when higher filler contents were used. Moreover, the compatibilized PLA/CNC with polyethylene glycol (PEG), as plasticizer, exhibited the smallest fiber diameters, reaching a value (49 ± 8 µm) less than 10 µm when compared to the neat PLA and this is due to the plasticizing effect of PEG, which tended to reduce the filament viscosity. The melt-spinning of PLA, followed by coating with CNCs and polyvinyl acetate (PVAc) to improve the hydrophilicity as well as to create a roughened surface for tissue engineering applications, was reported by Hossain et al. [84]. This allowed the addition of higher CNCs loadings (>85 wt %) and promoted adhesion between the CNCs and PLA through PVAc as a binder. Figure 5a shows schematic the representation of the coating method employed by using a syringe needle. The surface roughness of the fibers increased with an increase in the CNCs content in PVAc when compared to pure PLA (Figure 5b). At higher CNCs loadings, i.e., 95 wt %, uneven attachment of CNCs on the fiber surface was observed due to insufficient amount of PVAc binder present in the coating materials to provide homogeneous coverage, leading to CNCs clustering. Cytocompatibility studies by using NIH-3T3 mouse fibroblast cells, cultured onto CNCs-coated PLA surface, exhibited better cell adhesion when compared to PLA fibers.

### 3.4. Other Methods

#### 3.4.1. Three-Dimensional (3D) Printing

Additive manufacturing, also known as rapid prototyping or three-dimensional (3D) printing, has received tremendous interest from different fields, such as construction, aerospace, healthcare and many others [85]. This technique offers rapid fabrication of high-resolution, complex and reproducible constructs, through a computer-aided design (CAD). Among other 3D printing techniques, fused deposition modeling (FDM) by nozzle-deposition-based extrusion, employing different polymers, e.g., acrylonitrile butadiene styrene, PLA, poly(ε-caprolactone) (PCL), polyvinyl alcohol (PVAc), polymamides (nylon), etc., has shown a great potential.

**Figure 6:**
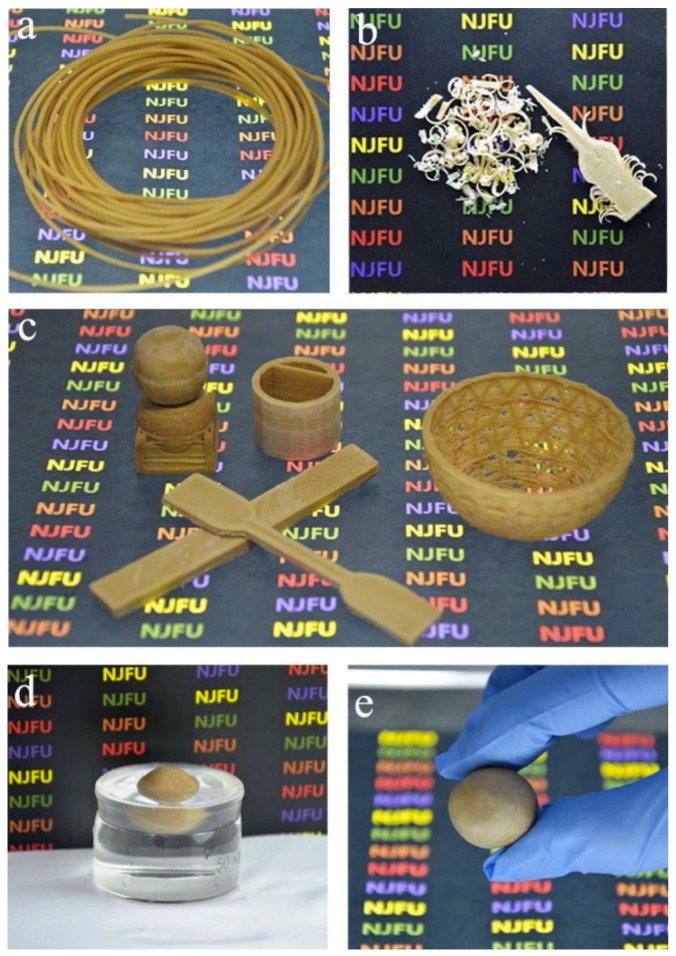
Photos of the 3D printed objects with the MNC/PLA composites: the MNC/PLA composite 3D printing wire rods (**a**); the 3D printed material subjected to planing and sawing (**b**); samples of 3D printed objects, including double-balls standing on the shelf, buckets, half-baskets, and sticks in elongated and dumbbell shape that were used for the testing of mechanical properties (**c**); the 3D printed solid ball floated on the water (**d**); and the 3D printed solid ball with 2 cm diameter (**e**). Reprinted with permission from [86].

Murphy and Collins [64] investigated the fabrication of a novel MCC reinforced PLA, i.e., fully degradable biocomposites for 3D printing applications. To achieve good dispersion of the MCC in PLA, a two-step process was adopted: a PLA/MCC film casting, followed by an extrusion process. They selected 1 and 3 wt % unmodified cellulose for printing by using a fusion deposition modeling of a 3D printer. It was demonstrated that, by choosing biomedical scaffolds prototypes, it was possible to produce constructs by using CAD software with a diameter of 15 mm and a height of 1 mm, having porous structure achieved by selecting a 60% fill density in the FDM software. It is reported that, for the use of plasticizer (polyethylene glycol) and the surface modification (silane coupling agent KH-550) of the cellulose, nanomaterials are essential to enable the 3D printable material [86]. In this study, a combination of micro- and nano-cellulose was obtained by mechanical disintegration, followed by solvent exchange in dichloromethane (DCM) to obtain their combination with PLA solution (in DCM) and polyethylene glycol (PEG6000) solution (in DCM). The composite suspension obtained was then dried and extruded into 3D printable wire rods, in which various types of objects were printed, such as double-balls standing on a shelf, sticks, half baskets, buckets, and single balls (Figure 6a). The authors reported that the composite composed of 30% cellulose, 65% PLA and 5% PEG600 had acceptable melt flow rates for 3D printing. The resulting 3D printed product had valuable attributes, such as being mechanically strong (elongation at break of 12%, tensile strength of 59.7 MPa and flexural strength of 50.7 MPa), lightweight and waterproof. The latter was justified by there being no significant weight increase after soaking the ball in water for 24 h (Figure 6d).

#### 3.4.2. Injection Molding

Injection molding, often employed for molding semi-finished samples, has also been used as preparation method for PLA/CNM composite materials. Injection molding of PLA/phenylphosphonic acid zinc (PPA-Zn) composite was carried out by injecting the samples into a pre-heated mold at different mold temperatures (40, 80 and 95 °C) and holding times (10, 20, 30, 60 and 120 s) [87]. It was reported that, when samples were ejected from the mold at a holding time of 10 s, only PLA/PPA-Zn/NFC composite could be obtained without distortion, while neat PLA and the PLA/PPA-Zn and PLA/NFC composites were all deformed. This was attributed to the high rigidity of PLA/PPA-Zn/NFC, when compared to other composites.

#### 3.4.3. Solid State Drawing

**Figure 7:**
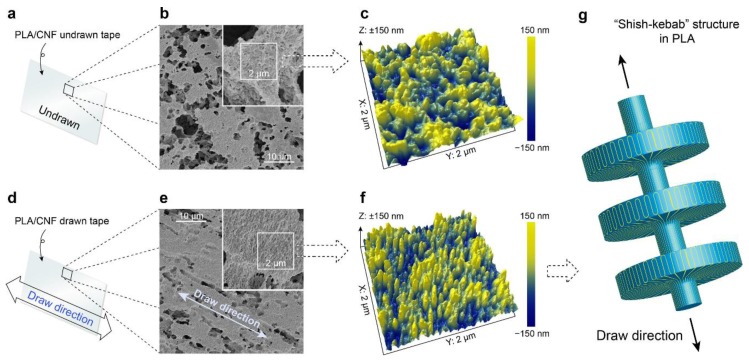
(**a**) Schematic representation of undrawn tape and (**b**,**c**) SEM and AFM images of undrawn tape respectively after etching; and (**d**) schematic representation of tape indicating drawing direction and (**e**,**f**) SEM and AFM of drawn oriented tape respectively after etching; and (**g**) Penning’s model representing “shish-kebab” structure. Reprinted with permission from [88].

Solid-state drawing can be regarded as post-treatment of the composite material. In this regard, the composite is often prepared by melt compounding technique (e.g., extrusion, followed by melt compression to form films) and the films obtained were cut into rectangular strips and then exposed to uniaxial solid-state drawing by using a tensile tester, equipped with a temperature chamber (Figure 7) [88,89]. It was recognized that the solid-state drawing, effectively orientated both the polymer and the reinforcements and, hence, leading to a better organized molecular structure with increased crystallinity, which, in turn, affected the mechanical and thermal properties of the resulting composite products, depending on the draw temperature, draw speed, draw ratio and the amount of reinforcement [89]. This process enabled the alignment of the macromolecular chains in the perpendicular to the drawing direction to form a “shish-kebab” because of the crystallization induced by deformation (Figure 7). Shish type crystallization orientates in parallel to the drawing direction and the kebab type crystallization grows vertically on the surface (Figure 7) [88,89]. It was also reported that, irrespective of the draw temperature or speed, the thickness of the nanocomposite tapes, decreased after drawing [88].

## 4. Mechanical Properties

### 4.1. Cellulose Nanocrystals

Numerous researchers have investigated the reinforcement ability of cellulose nanomaterials for PLA, as summarized in Table 5 [82]. Among all other factors, the preparation method, filler content, and interaction between the filler and matrix were found to have important effects on the properties of the resulting PLA/CNMs composite materials [90]. Oksman and co-workers [82] investigated the effect of cooling on the mechanical properties of PLA/CNC nanocomposites, plasticized with triethyl citrate. Extruded composites were compression-molded and then cooled, either in air (fast cooling) to avoid crystallization or inside the metal plates (slow cooling) to allow crystallization. The fast-cooled samples displayed yielding point, followed by strain softening and strain hardening, indicating their ductile nature. The fast-cooled samples, however, did not show necking, but showed stress whitening, indicating crazing. It was reported that the yield strength and Young’s modulus of the fast-cooled samples improved by 316% (from 3.7 to 15.4 MPa) and by 267% (from 0.3 to 1.1 GPa), respectively, with the addition of only 1 wt % CNCs. On the other hand, the addition of CNCs did not significantly affect the ultimate strength and the elongation at break for the fast-cooled samples. In the case of the slow cooled samples, the ultimate strength increased by 26% (from 15.8 to 19.9 MPa) by the addition of CNCs. The Young’s modulus increased by 50% (from 0.6 to 0.9 GPa), while the toughness (from 16 to 5.6 MJ/m^3^) and the elongation at break decreased (from 91% to 30%) in comparison with that of the plasticized PLA, due to their higher degree of the crystallinity, as confirmed by DSC. Elsewhere, it was reported that coating melt-spun PLA with PVAc-CNCs did not significantly influence the tensile properties [84]. PLA/PVAc, PLA65 wt %-CNCs20*w*/*v*-PVAc35 wt %, and PLA75 wt %-CNCs20*w*/*v*-PVAc25 wt % revealed 9%, 7% and 0.5% decreases and PLA85 wt %-CNCs 20*w*/*v*-PVAc15 wt % and PLA95 wt %-CNCs 20*w*/*v*-PVAc5 wt % exhibited 4% and 0.5% increases in tensile strength when compared with the uncoated PLA fibers (tensile strength 207 MPa). A significant improvement in the tensile modulus, however, for PLA65 wt %-CNCs20*w*/*v*-PVAc35 wt %, PLA75 wt %-CNCs20*w*/*v*-PVAc25 wt % and PLA85 wt %-CNCs20*w*/*v*-PVAc15 wt % fiber (33%, 43% and 45%, respectively) was observed when compared to the uncoated PLA fibers (tensile modulus 4.9 GPa) indicating the significant influence of CNCs (tensile modulus of 105 GPa) deposited on the fiber surface.

In summary, the tensile modulus and tensile strength of CNC/PLA composites increase with increase in the CNC content, however, at the expense of elongation at break [90]. However, the surface modification of CNC or the use of a coupling agent improves the interaction between CNC and PLA, which improves the stress transfer from the stiffer CNC to the polymer matrix, and this enhances the elongation at break and the toughness of the resulting material. The use of low contents of CNC, i.e., 1 wt % has no significant influence on the elongation at break (or toughness) and the tensile strength of the PLA/CNC composite materials, but, in some instances, increases the Young’s modulus [90]. It is worth mentioning that there is an optimal concentration of CNCs in PLA to enhance the resulting mechanical properties, beyond which the converse prevails. This is generally attributed to the interaction between adjacent CNCs to form strong network, resulting from the hydrogen bonding, known as “percolation effect”. It is believed that such network is responsible for significant improvements in the mechanical properties. Further increase in CNCs contents leads rather to agglomeration, thereby resulting in local stress concentrations and reduced strain to failure, hence adversely affecting the resulting mechanical properties [90].

### 4.2. Cellulose Nanofibers (CNF)

Several researchers have reported on the use of CNF to reinforce PLA [67,71,92,94,95,96]. In general, the Young’s modulus and the tensile strength increased with increasing CNF content [67,71,94]. On the other hand, the elongation at break (and hence the toughness), usually decrease with the CNF content. Recent study by Ozcan and co-workers [71] used epoxidized soybean oil (ESO) as plasticizer to compensate for the elongation at break in the presence of CNFs as the reinforcing phase. From their results, the combination of CNF and ESO in PLA matrix led to a composite with tensile strength similar to neat PLA, whereas the modulus increased with more than 20% over that of neat PLA. However, the ductility and toughness were at least three-fold higher than those of neat PLA. Further increase in CNF content to 20 and 30 wt %, however, did not exhibit any obvious improvement in strength, modulus or strain when compared to the same samples without the addition of 5 wt % ESO. This was ascribed to the CNF percolation, which became more dominant and allowed force transfer to occur between the CNF and the polymer matrix, such that the effect of ESO became negligible. The mechanical properties of such ternary systems depend on the type of plasticizer and its interaction with the cellulose nanofibers. Elsewhere, it was reported that the addition of 1 wt% of CNF increased the elongation at break and the work of fracture by 27% and 57%, respectively, when compared to PLA/glycerol triacetate (GTA, plasticizer), due to the plasticization of the nanofibers, together with the slippery effect of the CNFs in the PLA matrix [92]. 

**Figure 8:**
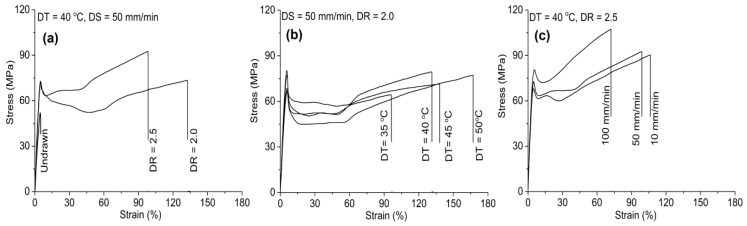
Typical stress-strain curves of the nanocomposite tapes: (**a**) different drawing ratio (DR) drawn at 40 °C with drawing speed (DS) of 50 mm/min; (**b**) DR 2.0 drawn at different temperatures with DS of 50 mm/min; and (**c**) DR 2.5 drawn at 40 °C with different speeds. Reprinted with permission from [88].

The preparation method and the fiber morphology/size play a significant influence on the mechanical properties of the ensuing composite. These factors affect the interaction between the filler and the matrix as well as the filler distribution, which greatly affects the stress transfer between the stiff filler and the polymeric matrix. The effect of post-treatment (crystallization) on the mechanical properties of the extruded composites were reported by Suryanegara, Nakagaito and Yano [95]. After compression molding, the samples were either immediately quenched in liquid nitrogen to obtain a fully amorphous state (amorphous samples) or melt-crystallized on a hot press at 100 °C for an hour to obtain a highly crystallized solid structure (crystallized samples). For amorphous samples, the addition of 20 wt % NFC improved the modulus from 3.3 to 5.2 GPa and the tensile strength from 58 to 70 MPa, while the strain at break reduced from 7% to 2%. This was attributed to the stiff fibers when compared to the PLA in the case of modulus, whereas the increase of strength was associated with good interfacial adhesion between the matrix and the fibers. Crystallization of PLA increased the tensile modulus (from 3.3 to 4.0 GPa) and the strength (from 50.2 to 60.9 MPa), while strain at break decreased from 7% to 3%. However, crystallized composite with 20 wt % NFC improved the modulus from 5.2 to 5.7 GPa, whereas the strain at break and the tensile strength were similar to those of the amorphous samples. This was attributed to the crystallization that occurred, leading to the embrittlement of the PLA. Two different preparation methods (viz. solvent casting, followed by kneading by using a twin rotary roller mixer (solvent method) and the addition of CNFs suspension, directly into the melted PLA (direct method)) and the filler morphology were investigated by Iwatake, Nogi and Yano [68]. In the case of the filler morphology, they compared three fillers, viz. microfibrillated cellulose (MFC, from Daicel Chemical Industries, Tokyo, Japan), needle-leaf bleached kraft pulp (NBKP) and refiner-treated NBKP (eight passes), as reinforcements for PLA. For the solvent method, the Young’s modulus and tensile strength increased from 3.4 GPa and 56.2 MPa to 4.3 GPa and 66.0 MPa, respectively, with the addition of 5 wt % of MFC. On the other hand, the composites made by direct mixing exhibited no improvement in Young’s modulus, whereas the yield strain was smaller than that of pure PLA, resulting in the reduction of the tensile strength by 10%. This was attributed to the difference in the dispersion of the fibers with solvent method, resulting in the uniform dispersion of MFC, while many clusters were observed for composite made by the direct mixing. In the case of the filler morphology, solvent method was used to prepare the composites. The pulp had smooth surface with 30–50 µm in diameter, the refiner-treated pulp had a fibrillated surface with diameter similar to the pulp and the MFC was completely disintegrated into nano to submicron wide fiber-forming network. The incorporation of pulp (5 wt %) slightly increased the Young’s modulus, but reduced the yield strain and strength by 30% and 15%, respectively. Refiner-treated pulp (5 wt %), however, improved both the Young’s modulus and the yield strain, which resulted in strength increase by 10%, while significant improvements in properties were exhibited by MFC (5 wt %)-reinforced PLA with Young’s modulus reaching an increment of 25% (without a reduction in the yield strain) and strength was improved by 20% when compared to neat PLA. This was associated with the formation of rigid network, resulting from strong interaction between the adjacent cellulose fibers by hydrogen bonding (percolation effect). MFC created a fine network even at low filler content (5 wt %), which restrained polymer deformation. Elsewhere in the literature, it was reported that the surface modification of CNFs with silane led to a yield strain increment of 25% when compared to pure polymer, due to the strong interaction between the two components of the composite [65]. However, the increase in CNF content led to a decrease in strain, because of the major role played in response to uniaxial stress by CNFs, which generated more dislocations in the composite. However, at high filler concentrations, the more agglomeration of the filler was observed, resulting in the deterioration of the tensile stress transfer between the matrix and the filler, which can lead to a more brittle structure. The effect of solid drawing at different drawing ratio, temperature and speed on the mechanical properties was reported by Singh et al. [88]. The stress–strain behavior of the tapes drawn below the PLA glass transition temperature (*T*_g_) (i.e., 35 °C), exhibited typical deformation behavior with elastic region, followed by the yield and subsequent drop in strength with strain softening (Figure 8). The tapes drawn in temperatures above *T*_g_ (40 °C) showed a decrease in the tensile strength and a weaker strain softening, while the tapes drawn at higher temperatures (45 and 50 °C) displayed a rubber-like behavior with a considerably lower strength, less prominent yield and strain softening, as shown in Figure 8a,b. The difference in the strain softening was attributed to the fact that, during drawing, there was the formation and growth of cavities at different draw temperatures. In the case of the speed of drawing at 40 °C, the highest drawing speed (100 mm/min) led to a significantly increased yield stress with pronounced strain softening and uniform drawing, while the lowest draw speed (10 mm/min) resulted in rubber-like behavior and a non-uniform tape resulted, while the yield and strain softening regions were less prominent. In short, the sample obtained from a draw ratio of 2.5, temperature of 40 °C and draw speed of 100 mm/min exhibited maximum Young’s modulus of 2.1 GPa and tensile strength of 102 MPa, which were 50% and 100% higher than those of the undrawn sample, respectively (Figure 8(c)). The sample obtained from a draw ratio of 2.0, temperature of 50 °C and draw speed of 50 mm/min displayed the highest elongation at break and work at fracture, which were 26 and 60 times higher than those of the undrawn tape. 

Compression molding of stacked cellulose nanofibers sandwiched between PLA films was found to have improved the mechanical properties of the resulting composite material [97]. The sandwich composite composed of volume fraction (*V*f) ~65 vol.%, resulted in increases in the elongation at break from 1.6% to 6.2% and Young’s modulus from 3.9 to 9.5 GPa, while the tensile strength increased from 55 to 270 MPa when compared to the neat PLA [97]. Elsewhere, nanopaper-reinforced PLA composite laminates reinforced with 10 × 5 g/m^2^ (Laminate 1, *V*f = 39), 5 × 10 g/m^2^ (Laminate 2, *V*f = 48), 2 × 25 g/m^2^ (Laminate 3, *V*f = 50) and 1 × 50 g/m^2^ (Laminate 4, *V*f = 53) were found to have excellent reinforcing ability of PLA [98]. Tensile moduli of between 10.5 and 11.8 GPa and tensile strength of between 95 and 111 MPa were obtained. 

In summary, the Young’s modulus and the tensile strength increase with increasing CNFs content due to the stiffness of the CNFs and the adhesion between the two components. The smaller size of the fibers promote the interaction between PLA and CNFs and, thus, the mechanical properties. Similar to CNCs, CNF has a percolation threshold, after which the mechanical properties cannot improve further. This results from the formation of strong network via hydrogen bonding. The elongation at break often decreases with an increase in the CNFs concentration. This is due to the stiffening effect of the CNF, which can be compensated for by adding plasticizer. The interaction between plasticizer and CNFs, however, has to be limited, such that it cannot promote slippage between fibers and, thus, adversely affect the mechanical properties. Of all the melt compounding processing methods, compression molding gives better mechanical properties with regards to higher content of cellulose materials that can be incorporated.

## 5. Dynamic Mechanical Properties

### 5.1. Cellulose Nanocrystals (CNC)

**Figure 9:**
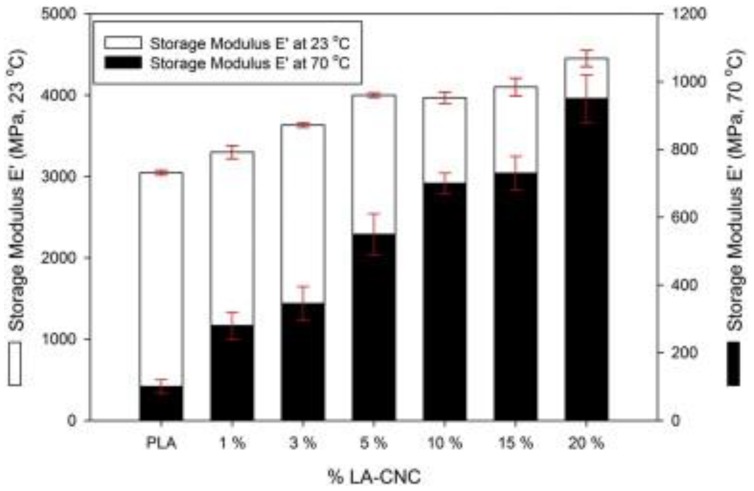
Storage modulus for PLA/LA-CNC nanocomposites at 23 and 70 °C. Reprinted with permission from [52].

Dynamic mechanical analysis (DMA) can elucidate the reinforcing effect of the cellulose nanomaterials by measuring the storage modulus as a function of temperature in both the glassy and rubbery states. The interaction between PLA and cellulose nanomaterials as well cellulose nanomaterial concentration was studied by Spinella et al. [52]. They found that the addition of modified CNCs led to slight increments of the modulus in the glassy state (23 °C) and large increase was obtained in PLA’s rubbery zone (70 °C). In the glassy state, unmodified CNC (HCℓ-CNCs), chemically modified CNCs, viz., lactic acid (LA-CNCs) and acetate (AA-CNCs) storage moduli increased from 3300 ± 30 MPa to 4000 ± 25 MPa and 3676 ± 40 MPa, respectively. In the rubbery state, values of the storage modulus increased from 146 ± 30 MPa to 265 ± 35 MPa and 550 ± 65 MPa for HCℓ-CNCs, AA-CNCs and LA-CNCs-based composites, as shown in Figure 9. This was attributed to the inclusion of rigid filler and the enhanced interfacial compatibility and adhesion between the modified fillers and PLA.

Comparison of the storage modulus for PLA/CNC nanocomposites filled with 1 wt % of different acid-derived CNCs were conducted by Dhar et al. [99]. The extracted CNC displayed different morphologies and dimensions, which led to variable aspect ratio of: ~50, ~17, ~57 and ~24 for H_2_SO_4_, HCℓ, H_3_PO_4_ and HNO_3_ hydrolyzed CNCs. The addition of phosphoric acid hydrolyzed CNC into PLA significantly increased storage modulus (*E*’) values by 1.75 times when compared to 1.5 and 1.4 times for the hydrochloric and sulfuric acid hydrolyzed CNC, with the least being 1.01 times for nitric acid hydrolyzed CNC (all compared to neat PLA). This was attributed to stronger interfacial adhesion for phosphoric acid hydrolyzed CNC with PLA because of substitution of –OH groups with phosphate groups onto the CNCs, which led to the introduction of hydrophobic character along with the presence of high degree of inter-molecular hydrogen bonding. Stronger interaction enhances the transfer of elastic modulus of the CNC to the polymeric material effectively, thereby improving the storage modulus of the nanocomposites. On the other hand, the high aspect ratio of CNC-H_3_PO_4_ compared to other acid derived CNCs led to higher reinforcement efficiency at relatively low volume fractions resulting in improved stress transfer from CNCs to the PLA matrix. It was found that an increase in CNC loadings, i.e., from 1 to 3 wt %, drastically increased the complex modulus of the nanocomposites because of an increase in the hydrogen bonding fraction (FH-CO) values (representing the degree of the hydrogen bonding between the PLA matrix and the CNC). The increase in FH-CO values indicate that the hydrogen bonding improved and better uniform distribution was achieved of CNC-H_3_PO_4_ in the PLA matrix. Weak intermolecular hydrogen bonding for CNC-HCℓ and low aspect ratio was reported to result in a lower effective stress transfer and thus, a decrement in the complex modulus. However, the hydrophilicity of CNC-HNO_3_ and CNC-H_2_SO_4_ led to their irreversible agglomeration, which affected the morphology and elastic modulus of the resulting nanocomposites. This study indicates that a selection of appropriate hydrolyzing acids for the extraction of CNC has to be carefully considered with regard to the desired characteristics, which could further affect the performance of the nanocomposite materials. The reactive extrusion of PLA in the presence of dicumyl peroxide to afford grafting of PLA chains onto CNCs and a formation of C–C bond was found to overcome the irreversible agglomeration of H_2_SO_4_ acid hydrolyzed CNC, as reported by Dhar et al. [91]. This resulted in an increase in the elastic modulus when 2 wt % of CNC was incorporated into the system due to the formation of crosslinked structure and better interfacial adhesion with the polymer matrix. Elastic moduli values were significantly higher for the reactively grafted CNC than the ungrafted PLA/CNC composite over the whole temperature range, which justifies the formation of C–C bonds and hence, resulting in the effective transfer of CNC modulus to the PLA matrix. 

**Figure 10:**
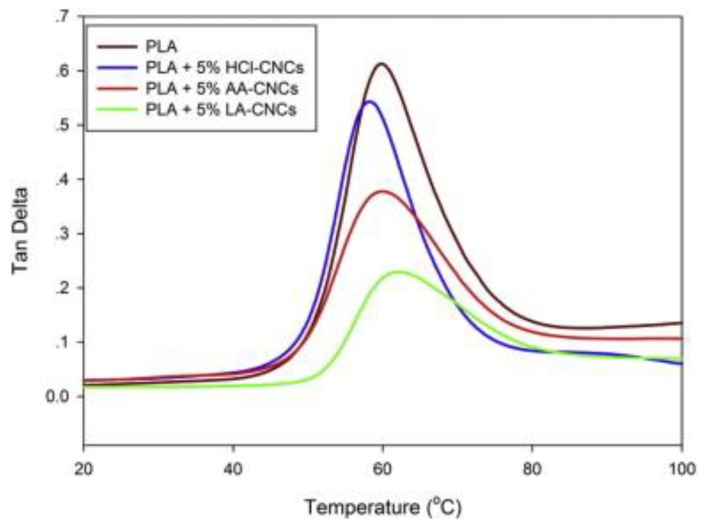
Tan Delta of modified and unmodified CNCs. Reprinted with permission from [52].

Surface topochemistry on the interaction between cellulose nanomaterials and PLA can be verified by tanδ (the damping factor) from DMA analysis, since it is related the polymer chains relaxations [52]. For instance, when there is strong interaction between PLA and the filler, the following changes will occur: (i) shifting in the peak maximum to higher temperatures; (ii) a decrease in the intensity at the peak maximum; and (iii) broadening of the transition. Elsewhere, CNCs that were chemically modified and included in lactate (LA-CNCs) and acetate (AA-CNCs) were compared with unmodified HCℓ-CNCs to investigate the surface topochemistry on their interaction with PLA [52]. The authors reported that at 5% HCℓ-CNCs, there was slight decrease (10%) in the tanδ peak and the peak temperature (59–58 °C), hence confirming poor adhesion, whereas, for AA- and LA-CNCs, 38% and 64% decreases were recorded at 5% loading (Figure 10). However, the maxima for the tanδ peaks for AA- and LA-CNCs were at 61 and 65 °C, indicating a stronger interaction between modified CNCs and PLA. It is worth mentioning that the maximum tanδ peak is often recognized as the glass transition of polymeric material. The maximum tanδ peak was found to decrease for the reactively extruded PLA chains, grafted onto CNCs by using dicumyl peroxide as radical initiator, even though a better interaction between CNC and PLA was achieved [91]. This was attributed to the crosslinked PLA-*g*-CNC gels, undergoing heat shrinkage at *T*_g_, which depends on the degree of branching.

### 5.2. Cellulose Nanofibers

The presence CNF was found to enhance the elastic modulus of the composite material, regardless of the surface functionalization of the CNF or the use of coupling agent/compatibilizer. This can be related to the higher stiffness of the cellulose nanofibers when compared to neat PLA. Meng et al. [71] reported that storage modulus of CNF increased (from 2680 MPa at 25 °C to 3079 MPa) with the addition of 10 wt % of CNF. They further reported that, despite the presence of epoxidized soybean oil (ESO) as plasticizer, the storage moduli further increased with increase in the CNF content. The intensities of the tanδ peaks were also decreased with increases in the CNF contents and the glass transition temperatures increased due to the restriction of segmental chain mobility of the PLA matrix by CNFs.

Oksman and co-workers prepared PLA/CNFs by extracting CNFs from kenaf pulp, by mechanical grinding, followed by solvent exchange to acetone to enable the preparation of a master-batch, based on PLA and CNF in acetone and chloroform (9:1) to avoid the re-aggregation of the CNFs, during extrusion process [67]. The storage modulus was significantly higher for all composites than the neat PLA and the improvement was most significant at 5 wt % of CNF, due to CNF entanglement. The improvement was more pronounced at 70 °C, where the storage modulus reached a value of 2.5 GPa for PLA-CNF (5 wt %) when compared to 100 MPa for pure PLA. Such improvements, after the relaxation, forming a “plateau”, was related to the entanglement of the fibers, especially at higher content of CNF. The tanδ peak also shifted to higher temperature with increased in CNF content of 5% CNF-based composite, reaching 76 °C when compared to 70 °C for neat polymer matrix. Furthermore, tanδ peak intensity decreased with increase in CNF concentration when compared to the neat PLA, indicating the fact that very few polymer chains were involved in this transition. The increase in storage moduli and the shifting of tanδ was attributed to physical interaction between the polymer and the CNF reinforcement that restricted the segmental mobility of polymer chains in the vicinity of the nano-reinforcements. Crystallization of samples prepared by hot-pressing the composites for 1 h resulted in a storage modulus of ~1 GPa at 120 °C, indicating that the combination of NFC reinforcement and the crystallization of PLA contributed to better heat resistance of the composite, thereby showing its potential in applications for products that may be exposed to high temperatures [95]. The constant storage modulus above the glass transition temperate of PLA, i.e., from 70 °C to 120 °C, was reported by Iwatake, Nogi, and Yano [68]. In this regard, 10 wt % of CNF in PLA matrix exhibited such constant storage modulus, independent of the softening of PLA due to the cellulose fiber network that was interconnected by hydrogen bonds, thereby resisting the applied stress, regardless of the softening of PLA.

In summary, the presence of both CNF and CNC in PLA increases the storage modulus of the resulting composite materials. The higher reinforcing effect is observed at a concentration at which the CNMs can form a rigid network, resulting from the interaction between these cellulosic particles, via hydrogen bonding and this is dependent on the aspect ratio of the CNMs. This restricts the segmental mobility of PLA chains and, thus, the tanδ peak shifts to higher temperatures or its intensity decreases. The strong interaction between the filler and PLA improves the reinforcing effect of CNMs, especially at higher temperatures above the glass transition temperature of the PLA. The preparation method is significant to achieve better dispersion as well as the envisaged CNM reinforcing potential.

## 6. Thermal Properties

**Figure 11:**
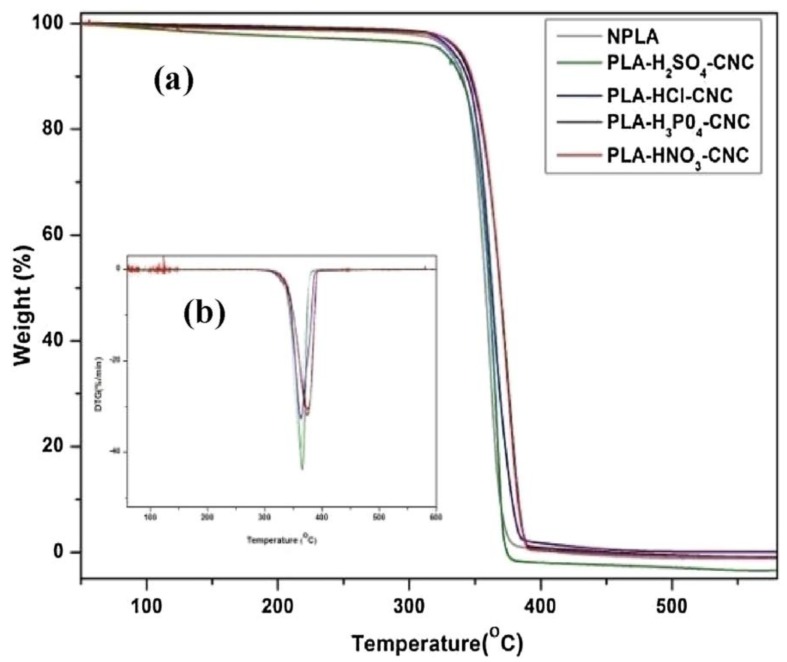
(**a**) Weight (%) versus temperature graph obtained from the TG data; and (**b**) DTG plots of PLA with different acid hydrolyzed CNC nanocomposite at β = 10 °C/min. Reprinted by permission from [100].

Thermal stability of the materials is one of most important aspects, especially when considering the heat involved during their preparation, when employing thermoplastic processing techniques. In general, in the presence of cellulose, nanomaterials were found to decrease the thermal stability of the resulting composite materials [63,86]. Depending on the processing technique, the material thermal stability has to withstand or show no thermal degradation below the desired processing temperature. For instance, Wang et al. [86] reported that the PLA-cellulose nanomaterial prepared had no weight loss below 260 °C, which afforded their applications in the 3D printing environment. The chemical modification of the cellulose nanomaterials can also improve the thermal stability of the resulting composite materials due to the strong interaction, which allows the polymer to be protected from heat. Elsewhere, it was reported that the reduction in the thermal stability was found to be less pronounced for the chemically modified materials, which was attributed to a strong interaction and adhesion [52]. On the other hand, the cellulose nanomaterials extraction process can influence the thermal properties of the resulting composite material due to the presence of the functional groups introduced by these treatments. A detailed study on the effect of the CNC fabricated by using different acids (i.e., hydrochloric, nitric, phosphoric and sulfuric acid) on the thermal behavior of the PLA was carried out by Monika, Dhar and Katiyar [100]. Freeze-dried CNC extracted by H_2_SO_4_, H_3_PO_4_, HNO_3_ and HCℓ, were added to PLA matrix (labeled PLA-H_2_SO_4_-CNC, PLA-H_3_PO_4_-CNC, PLA-HNO_3_-CNC and PLA-HCℓ-CNC, respectively) and melt-mixed by using a micro-twin-extruder at 180 °C and a screw speed of 50 rpm. The thermal stability followed the order: PLA-HNO_3_-CNC > PLA-H_3_PO_4_-CNC > PLA-HCℓ-CNC > PLA-H_2_SO_4_-CNC (Figure 11). The lowest thermal stability of H_2_SO_4_ hydrolyzed CNC-based composites was related to the presence of sulfate groups (〖SO〗^4−^) onto the CNC that may have catalytic effect on the nanocomposite chain of PLA, thereby leading to increased thermal decomposition rate. The improved thermal stability on other acids was ascribed to the stable bond formation between the carbon and substituted anion (for example, the bond strength C-N > C-P) or the thermal behavior of the substituted anion (for example, thermal stability 〖Cl〗^−^ > 〖SO〗^4−^) on the CNC surface. The results clearly indicate that the thermal behavior of the composites can be influenced by the extraction method or hydrolyzing acid employed. Similar observations were reported in another study by Katiyar and co-workers [99]. In this study, it was found that the thermal stability of PLA/CNC nanocomposites followed the same order: PLA-HNO_3_-CNC > PLA-H_3_PO_4_-CNC > PLA-HCℓ-CNC > PLA-H_2_SO_4_-CNC. It was concluded that the thermal properties for the polymer-CNC nanocomposites fabricated were dependent on the inherent thermal properties of the different acid-derivatized CNCs. To resolve the issue of thermal stability of CNC extracted by using H_2_SO_4_ acid hydrolysis, the improvement of the interaction between CNCs and PLA allows the PLA matrix to encapsulate CNC-H_2_SO_4_, such that the thermal stability can be improved. Dhar et al. [91] grafted PLA chains onto CNC surface, although there was C–C bond formation in the presence of dicumyl peroxide (DCP), as a radical initiator. It was reported that grafting PLA onto CNCs in the presence of DCP through reactive extrusion led to an improvement in thermal stability by ~12 °C and ~5 °C for onset degradation temperature (Tonset) and the temperature at which 50% (T50%) mass loss was observed, respectively. This was attributed to the formation of PLA encapsulated CNCs during reactive extrusion, which masks sulfate and hydroxyl groups of CNCs, thereby delaying their degradation process. However, the formation of C–C bond between PLA and CNCs led to enhanced thermal stability since higher activation energy is required to break such bridged linkages.

## 7. Future Remarks and Conclusions

Thermoplastic processing of PLA/CNMs has a promising potential for large-scale production of green composites towards various applications, which include packaging and biomedical fields. The utilization of CNMs, viz. CNCs and CNFs, as fillers renders efficient and attractive route to improve the properties of PLA, while preserving the biological and environmental advantages, i.e., biodegradability, cytocompatibility, compostability and renewability associated with PLA matrix.

Based on this review work, several conclusions on the preparation and properties of PLA/CNMs composite can be made:

Different factors, such as functionalization of cellulose nanomaterials and processing techniques to produce PLA/cellulose nanomaterials composites (viz., compression molding, melt compounding, melt spinning, injection molding and solid-state drawing) play a significant role in the overall properties of the PLA/CNMs composites. The preparation method, filler content and type, and interaction between the filler and matrix have paramount effects on the properties of the resulting PLA/CNMs composite materials. Functionalized PLA nanocellulose composites show better properties than their unmodified counterparts due to improved dispersion and interaction with PLA matrix. The filler content has an optimal concentration in the PLA matrix for the purpose of enhancing the resulting properties of the nanocomposites, after which the opposite trend prevails.

Compression molding enables an opportunity to incorporate large quantities of CNMs and again provides sufficient time for CNM filler–filler interaction, which enhances the overall composite properties when compared to other thermoplastic processing techniques. Secondary processing techniques, such as solid drawing and 3D-printing, further open doors for PLA composites for advanced applications. The presence of the CNMs enhances the mechanical, thermal and thermomechanical characteristics of the resulting PLA composites, which can allow their applications in various fields. The extraction process of CNMs plays a major role in the ensuing composite properties, since it controls their surface groups, hence the interaction with PLA. Furthermore, the improvement of the interfacial interaction between PLA and CNMs is important to achieve the desired properties. The use of other functionalization routes, such as employing biopolymers, e.g., PHB, serves as promising route not only to facilitate interaction between PLA and CNMs, but their contribution in improving the toughness of the composites, while preserving the biological and environmental properties.

The possibility of choosing between CNF and CNC allows these nanomaterials to have a bright future, making the filler not only feasible to produce composite with the desired overall properties, but to reduce the overall price of the resulting composites. The CNFs serve as fillers of choice since it is easier to extract when compared to CNCs, but they still suffer the same drawback when it comes to dispersability and compatibility. Moreover, the filler–filler interaction of both fillers to form an inter-network via hydrogen bonding still controls the mechanical properties enhancement of the resulting composite products.

In the future, the use of green compatibilizers to improve the dispersion and interfacial adhesion of PLA and CNMs would further enhance their performance. The development of an advanced single suitable and effective method to incorporate CNMs into PLA without losing the CNMs dispersed state (as in solution casting) is essential. Moreover, the addition of a second nanofiller can serve as an alternative route to promote interfacial adhesion and the overall properties, and to offer the resulting composites new opportunities with additional functionality.

## Figures and Tables

**Table 1:** Physical properties of PLA.

Polymer	Elastic Modulus (GPa)	Tensile Strength (MPa)	Elongation at Break (%)	*T*_m_ (°C)	*T*_g_ (°C)
Stereo complexed polylactic acid (PLA)	8.6	0.88	30	220–230	65-72
Syndiotatic PLA	-	-	-	151	34
Poly(l-lactide) PLLA	2.7–4.14	15.5–150	3.0–10	170–190	55–65
Poly(dl-lactide) PDLLA	1.5–1.9	0.04–0.05	5–10	170–190	50–60

**Table 2:** Cellulose nanomaterials (CNMs) properties.

Cellulose Source	Extraction Process	Length (nm)	Width (nm)	Aspect Ratio	*E*_A_ (GPa)	*E*_T_ (GPa)	Refs.
Tunicates	H_2_SO_4_	100–300	5–10	1–150	150 ± 28.8 ^a^	-	[24,25]
Green algae	Hydrobromic acid	390–580	10–25	50–100	-	-	[26]
Red algae	H_2_SO_4_	240–350	2–13	35–60	-	-	[27]
Cotton	-	100–150	10	20–70	105, 57 ^a^	-	[28]
Wood	H_2_SO_4_	50–300	2–8	20–50	110–200 ^a^	11–57 ^a^	[24]
Bacteria	H_2_SO_4_	100–1000	10–50	15–100			[24]
Bacteria	Sonication	-	35–90		78 ± 17 ^a^		[29]
Bacteria	NaOH				114 ^c^	-	[30]
Bacteria	HCℓ	160–420	15–25	7–23	-	-	[31]
NFC	Homogenization	-	-	-	65^b^	10–20 ^b^	[32]
NFC	TEMPO	1000	10–40	100–150	145.2 ± 3.3 ^a^	-	[25]
MCC	-	-	>2000		25 ± 4 ^c^	-	[33]

^a^ Atomic Force Microscopy (AFM); ^b^ High-Resolution Transmission Electron Microscopy (HR-TEM) micromechanics; ^c^ Raman (*E*_A_ elastic modulus in axial direction, and *E*_T_ elastic modulus in transverse).

**Table 3:** Selected studies on surface modifications of CNMs for PLA composites.

Type	Extraction Process and Source	Functionalization Method	Refs.
**CNCs**	Microcrystalline cellulose by acid hydrolysis	PLLA grafting using surface-initiated ring opening polymerization	[57]
	Microcrystalline cellulose by sulfuric acid hydrolysis	Glycidyl methacrylate (GMA) grafting	[58]
	Linter by sulfuric acid hydrolysis	PCL grafted via ring-opening polymerization under microwave irradiation	[56]
	Scoured cotton by HCℓ hydrolysis	One-pot Fischer esterification approach was adopted to esterify CNCs with lactic acid	[52]
	Bamboo particles by sulfuric acid hydrolysis	Salinization of CNCs by using triethoxysilane (A-151)	[55]
	Cotton pulp by mild H_2_SO_4_	Carboxylation using potassium permanganate and oxalic acid as oxidizing and reducing agent, respectively	[63]
**CNFs**	Microcrystalline cellulose was subjected to high-speed homogenizer	Acetylation using rice bran oil (RBO)	[54]
	Bamboo pulp pre-treated by 2,2,6,6-tetramethylpiperidine-1-oxy radical (TEMPO)-mediated oxidation by using a TEMPO/NaBr/NaClO system to facilitate disintegration by using a high-pressure homogenizer	Carboxylation of CNF via TEMPO oxidation	[61]
	Titanate coupling agent was dissolved in tetrahydrofuran (THF, 10 g) and introduced into 15 wt % of MCC in (THF)	The hydroxyl groups react with the monoalkoxy and neoalkoxy to form monomolecular layer	[64]
	Bleached pulp was homogenized with 30 passes at ~1000 bar and treated with 3-amino triethoxysilane (ATS)	Salinization using 3-amino triethoxysilane (ATS)	[65]

**Table 4:** Selected studies, based on thermoplastic processing techniques of PLA/cellulose nanocomposites.

Formulation	Pre-Processing Method	Processing Method	Post-Processing	Highlights	Refs.
PLA/PVAc-GMA/CNC 67/30/3	Polymerization of PVAc by using ammonium cerium (IV) nitrate as initiator and functional by grafting Glycidyl methacrylate (GMA), then mixed with CNC suspension followed by drying	Extrusion	-	Effective dispersion of CNCs was achieved	[69]
PLA/CNC-*g*-PLLA	CNC-PLLA grafted by surface-initiated ring opening polymerization (SI-ROP) of l-lactide	Extrusion	-	Smooth and uniform surface confirmed good dispersion of CNC in the matrix	[57]
PLA/CNC	-	Melt spinning	-	Surface roughness increased with an increase in content of cellulose nanocrystals	[70]
PLA/CNF	-	Melt-mixer	Compression molding	A morphological study with SEM revealed a good dispersion of CNFs as confirmed by well-distributed fiber “pull outs” with no visible aggregates	[71]
PLA/CNF	Casein protein as compatibilizer	Compression molding	-	The presence casein significantly improved interfacial adhesion (compatibility)	[72]
PLA/CNC	Esterified with hexanoic and dodecanoic	Melt spinning	-	Hexanoic-treated CNC exhibited highest draw ratio due to improved compatibility	[8]

**Table 5:** Tensile properties of thermoplastic processed PLA/CNMs composites.

Thermoplastic processed PLA/CNMs			Tensile Properties			
Formulation	Surface Functionalization	Processing Method	Tensile Strength (MPa)	Modulus (GPa)	Elongation (%)	Refs.
PLA/CNC 98/2	Hexanoic acid	Extrusion and melt spinning	141	5.73	17.0	[8]
PLA/CNC 98/2	Dodecanoic acid	Extrusion and melt spinning	123	5.72	38.4	[8]
PLA/CNC 99/1	Dicumyl peroxide as a radical initiator for reactive grafting of PLA chains onto CNCs	Extrusion	51	22.5	1.9	[91]
PLA/CNC 99/1	-	Extrusion	56.2	1.3	6.4	[90]
PLA/CNC 97/3	-	Extrusion	56.2	1.4	5.5	
PLA/CNC 95/5	-	Extrusion	54.9	1.4	4.9	
PLA/CNC 99/1	-	Solution casting + extrusion + melt spinning	52	2.5	13	[70]
PLA/CNC 97/3	-	Solution casting + extrusion + melt spinning	49	2.7	10	
PLA/CNF 95/5	-	Solvent casting + Extrusion	71.2	3.6	2.7	[67]
PLA/CNF/glycerol triacetate (GTA) 79/1/20	-	Peristaltic pump feeding (CNF+GTA) + Extrusion	28.8	0.8	31.1	[92]
PLA/CNF 35/65	-	Compression molding	121	12.4	3.4	[93]
PLA/CNF 30/70	-	Compression molding	121	13.4	2.3	
PLA/CNF 66/34	-	Compression molding	105	12.7	2.5	
PLA/CNF 28/72	-	Compression molding	95	13.6	1.6	
PLA/1 wt% CNF	1 wt% casein animal protein as compatibilizer	Compression molding	78	6.3	5.3	[72]

## References

[1] Chee W., Lim H., Huang N., Harrison I. (2015). Nanocomposites of graphene/polymers: A review. RSC Adv..

[2] Müller K., Bugnicourt E., Latorre M., Jorda M., Echegoyen Sanz Y., Lagaron J.M., Miesbauer O., Bianchin A., Hankin S., Bölz U. (2017). Review on the processing and properties of polymer nanocomposites and nanocoatings and their applications in the packaging, automotive and solar energy fields. Nanomaterials.

[3] Mtibe A., Mokhothu T.H., John M.J., Mokhena T.C., Mochane M.J., Mustansar Hussain C. (2018). Chapter 8—Fabrication and Characterization of Various Engineered Nanomaterials. Handbook of Nanomaterials for Industrial Applications.

[4] Scaffaro R., Botta L., Lopresti F., Maio A., Sutera F. (2017). Polysaccharide nanocrystals as fillers for PLA based nanocomposites. Cellulose.

[5] Mtibe A., Linganiso L.Z., Mathew A.P., Oksman K., John M.J., Anandjiwala R.D. (2015). A comparative study on properties of micro and nanopapers produced from cellulose and cellulose nanofibres. Carbohydr. Polym..

[6] Mtibe A., Mandlevu Y., Linganiso L.Z., Anandjiwala R.D. (2015). Extraction of Cellulose Nanowhiskers From Flax Fibres and Their Reinforcing Effect on Poly (furfuryl) Alcohol. J. Biobased Mater. Bioenergy.

[7] Foster E.J., Moon R.J., Agarwal U.P., Bortner M.J., Bras J., Camarero-Espinosa S., Chan K.J., Clift M.J., Cranston E.D., Eichhorn S.J. (2018). Current characterization methods for cellulose nanomaterials. Chem. Soc. Rev..

[8] Blaker J.J., Lee K.-Y., Walters M., Drouet M., Bismarck A. (2014). Aligned unidirectional PLA/bacterial cellulose nanocomposite fibre reinforced PDLLA composites. React. Funct. Polym..

[9] Arrieta M.P., Samper M.D., Aldas M., López J. (2017). On the use of PLA-PHB blends for sustainable food packaging applications. Materials.

[10] Fortunati E., Puglia D., Iannoni A., Terenzi A., Kenny J.M., Torre L. (2017). Processing conditions, thermal and mechanical responses of stretchable poly (lactic acid)/poly (butylene succinate) films. Materials.

[11] Hu Y., Daoud W.A., Cheuk K.K.L., Lin C.S.K. (2016). Newly developed techniques on polycondensation, ring-opening polymerization and polymer modification: Focus on poly (lactic acid). Materials.

[12] Reddy M.M., Vivekanandhan S., Misra M., Bhatia S.K., Mohanty A.K. (2013). Biobased plastics and bionanocomposites: Current status and future opportunities. Prog. Polym. Sci..

[13] Muller J., González-Martínez C., Chiralt A. (2017). Combination of poly (lactic) acid and starch for biodegradable food packaging. Materials.

[14] Oksman K., Aitomäki Y., Mathew A.P., Siqueira G., Zhou Q., Butylina S., Tanpichai S., Zhou X., Hooshmand S. (2016). Review of the recent developments in cellulose nanocomposite processing. Compos. Part A.

[15] Armentano I., Bitinis N., Fortunati E., Mattioli S., Rescignano N., Verdejo R., Lopez-Manchado M.A., Kenny J.M. (2013). Multifunctional nanostructured PLA materials for packaging and tissue engineering. Prog. Polym. Sci..

[16] Raquez J.-M., Habibi Y., Murariu M., Dubois P. (2013). Polylactide (PLA)-based nanocomposites. Prog. Polym. Sci..

[17] Tsuji H. (2005). Poly (lactide) stereocomplexes: Formation, structure, properties, degradation, and applications. Macromol. Biosci..

[18] Mokhena T.C., Jacobs N.V., Luyt A.S. (2018). Nanofibrous alginate membrane coated with cellulose nanowhiskers for water purification. Cellulose.

[19] Mokhena T., Jacobs V., Luyt A. (2015). A review on electrospun bio-based polymers for water treatment. Express Polym. Lett..

[20] Desmaisons J., Boutonnet E., Rueff M., Dufresne A., Bras J. (2017). A new quality index for benchmarking of different cellulose nanofibrils. Carbohydr. Polym..

[21] Mokhena T.C., Luyt A.S. (2014). Investigation of polyethylene/sisal whiskers nanocomposites prepared under different conditions. Polym. Compos..

[22] Lekha P., Mtibe A., Motaung T., Andrew J.E., Sitholè B.B., Gibril M. (2016). Effect of mechanical treatment on properties of cellulose nanofibrils produced from bleached hardwood and softwood pulps. Maderas Cienc. Tecnol..

[23] Motaung T.E., Mtibe A. (2015). Alkali treatment and cellulose nanowhiskers extracted from maize stalk residues. Mater. Sci. Appl..

[24] Sacui I.A., Nieuwendaal R.C., Burnett D.J., Stranick S.J., Jorfi M., Weder C., Foster E.J., Olsson R.T., Gilman J.W. (2014). Comparison of the properties of cellulose nanocrystals and cellulose nanofibrils isolated from bacteria, tunicate, and wood processed using acid, enzymatic, mechanical, and oxidative methods. ACS Appl. Mater. Interfaces.

[25] Iwamoto S., Kai W., Isogai A., Iwata T. (2009). Elastic modulus of single cellulose microfibrils from tunicate measured by atomic force microscopy. Biomacromolecules.

[26] Sucaldito M.R., Camacho D.H. (2017). Characteristics of unique HBr-hydrolyzed cellulose nanocrystals from freshwater green algae (*Cladophora rupestris*) and its reinforcement in starch-based film. Carbohydr. Polym..

[27] El Achaby M., Kassab Z., Aboulkas A., Gaillard C., Barakat A. (2018). Reuse of red algae waste for the production of cellulose nanocrystals and its application in polymer nanocomposites. Int. J. Biol. Macromol..

[28] Rusli R., Eichhorn S.J. (2008). Determination of the stiffness of cellulose nanowhiskers and the fiber-matrix interface in a nanocomposite using Raman spectroscopy. Appl. Phys. Lett..

[29] Guhados G., Wan W., Hutter J.L. (2005). Measurement of the elastic modulus of single bacterial cellulose fibers using atomic force microscopy. Langmuir.

[30] Hsieh Y.-C., Yano H., Nogi M., Eichhorn S. (2008). An estimation of the Young’s modulus of bacterial cellulose filaments. Cellulose.

[31] George J. (2012). High performance edible nanocomposite films containing bacterial cellulose nanocrystals. Carbohydr. Polym..

[32] Josefsson G., Tanem B.S., Li Y., Vullum P.E., Gamstedt E.K. (2013). Prediction of elastic properties of nanofibrillated cellulose from micromechanical modeling and nano-structure characterization by transmission electron microscopy. Cellulose.

[33] Eichhorn S., Young R. (2001). The Young’s modulus of a microcrystalline cellulose. Cellulose.

[34] Menon M.P., Selvakumar R., Ramakrishna S. (2017). Extraction and modification of cellulose nanofibers derived from biomass for environmental application. RSC Adv..

[35] Lee H.-R., Kim K., Mun S.C., Chang Y.K., Choi S.Q. (2018). A new method to produce cellulose nanofibrils from microalgae and the measurement of their mechanical strength. Carbohydr. Polym..

[36] Esa F., Tasirin S.M., Rahman N.A. (2014). Overview of Bacterial Cellulose Production and Application. Agric. Agric. Sci. Procedia.

[37] Picheth G.F., Pirich C.L., Sierakowski M.R., Woehl M.A., Sakakibara C.N., de Souza C.F., Martin A.A., da Silva R., de Freitas R.A. (2017). Bacterial cellulose in biomedical applications: A review. Int. J. Biol. Macromol..

[38] Reiniati I., Hrymak A.N., Margaritis A. (2017). Recent developments in the production and applications of bacterial cellulose fibers and nanocrystals. Crit. Rev. Biotechnol..

[39] Budhi Y., Fakhrudin M., Culsum N., Suendo V., Iskandar F. (2018). Preparation of cellulose nanocrystals from empty fruit bunch of palm oil by using phosphotungstic acid. IOP Conference Series: Earth and Environmental Science.

[40] Sadeghifar H., Filpponen I., Clarke S.P., Brougham D.F., Argyropoulos D.S. (2011). Production of cellulose nanocrystals using hydrobromic acid and click reactions on their surface. J. Mater. Sci..

[41] Tang Y., Shen X., Zhang J., Guo D., Kong F., Zhang N. (2015). Extraction of cellulose nano-crystals from old corrugated container fiber using phosphoric acid and enzymatic hydrolysis followed by sonication. Carbohydr. Polym..

[42] Filson P.B., Dawson-Andoh B.E. (2009). Sono-chemical preparation of cellulose nanocrystals from lignocellulose derived materials. Bioresour. Technol..

[43] Li B., Xu W., Kronlund D., Määttänen A., Liu J., Smått J.-H., Peltonen J., Willför S., Mu X., Xu C. (2015). Cellulose nanocrystals prepared via formic acid hydrolysis followed by TEMPO-mediated oxidation. Carbohydr. Polym..

[44] Du H., Liu C., Mu X., Gong W., Lv D., Hong Y., Si C., Li B. (2016). Preparation and characterization of thermally stable cellulose nanocrystals via a sustainable approach of FeCl3-catalyzed formic acid hydrolysis. Cellulose.

[45] Mihranyan A. (2011). Cellulose from cladophorales green algae: From environmental problem to high-tech composite materials. J. Appl. Polym. Sci..

[46] Chen Y.W., Lee H.V., Juan J.C., Phang S.-M. (2016). Production of new cellulose nanomaterial from red algae marine biomass Gelidium elegans. Carbohydr. Polym..

[47] Holland L.Z. (2016). Tunicates. Curr. Biol..

[48] Zhao Y., Zhang Y., Lindström M.E., Li J. (2015). Tunicate cellulose nanocrystals: Preparation, neat films and nanocomposite films with glucomannans. Carbohydr. Polym..

[49] Zhao Y., Li J. (2014). Excellent chemical and material cellulose from tunicates: Diversity in cellulose production yield and chemical and morphological structures from different tunicate species. Cellulose.

[50] Zhang T., Cheng Q., Ye D., Chang C. (2017). Tunicate cellulose nanocrystals reinforced nanocomposite hydrogels comprised by hybrid cross-linked networks. Carbohydr. Polym..

[51] Zhou L., He H., Li M.-c., Huang S., Mei C., Wu Q. (2018). Enhancing mechanical properties of poly (lactic acid) through its in-situ crosslinking with maleic anhydride-modified cellulose nanocrystals from cottonseed hulls. Ind. Crops Prod..

[52] Spinella S., Re G.L., Liu B., Dorgan J., Habibi Y., Leclere P., Raquez J.-M., Dubois P., Gross R.A. (2015). Polylactide/cellulose nanocrystal nanocomposites: Efficient routes for nanofiber modification and effects of nanofiber chemistry on PLA reinforcement. Polymer.

[53] Lin N., Huang J., Dufresne A. (2012). Preparation, properties and applications of polysaccharide nanocrystals in advanced functional nanomaterials: A review. Nanoscale.

[54] Kale R.D., Gorade V.G., Madye N., Chaudhary B., Bangde P.S., Dandekar P.P. (2018). Preparation and characterization of biocomposite packaging film from poly (lactic acid) and acylated microcrystalline cellulose using rice bran oil. Int. J. Biol. Macromol..

[55] Qian S., Sheng K., Yu K., Xu L., Lopez C.A.F. (2018). Improved properties of PLA biocomposites toughened with bamboo cellulose nanowhiskers through silane modification. J. Mater. Sci..

[56] Lin N., Chen G., Huang J., Dufresne A., Chang P.R. (2009). Effects of polymer-grafted natural nanocrystals on the structure and mechanical properties of poly (lactic acid): A case of cellulose whisker-graft-polycaprolactone. J. Appl. Polym. Sci..

[57] Lizundia E., Fortunati E., Dominici F., Vilas J.L., León L.M., Armentano I., Torre L., Kenny J.M. (2016). PLLA-grafted cellulose nanocrystals: Role of the CNC content and grafting on the PLA bionanocomposite film properties. Carbohydr. Polym..

[58] Pracella M., Haque M.M.-U., Puglia D. (2014). Morphology and properties tuning of PLA/cellulose nanocrystals bio-nanocomposites by means of reactive functionalization and blending with PVAc. Polymer.

[59] Bin Y., Yang B., Wang H. (2018). The effect of a small amount of modified microfibrillated cellulose and ethylene–glycidyl methacrylate copolymer on the crystallization behaviors and mechanical properties of polylactic acid. Polym. Bull..

[60] Immonen K., Lahtinen P., Pere J. (2017). Effects of Surfactants on the Preparation of Nanocellulose-PLA Composites. Bioengineering.

[61] Wu B., Geng B., Chen Y., Liu H., Li G., Wu Q. (2017). Preparation and characteristics of TEMPO-oxidized cellulose nanofibrils from bamboo pulp and their oxygen-barrier application in PLA films. Front. Chem. Sci. Eng..

[62] Wågberg L., Decher G., Norgren M., Lindström T., Ankerfors M., Axnäs K. (2008). The build-up of polyelectrolyte multilayers of microfibrillated cellulose and cationic polyelectrolytes. Langmuir.

[63] Zhou L., Li N., Shu J., Liu Y., Wang K., Cui X., Yuan Y., Ding B., Geng Y., Wang Z., Duan Y., Zhang J. (2018). One-pot preparation of carboxylated cellulose nanocrystals and their liquid crystalline behaviour. ACS Sustain. Chem. Eng..

[64] Murphy C.A., Collins M.N. (2018). Microcrystalline cellulose reinforced polylactic acid biocomposite filaments for 3D printing. Polym. Compos..

[65] Robles E., Urruzola I., Labidi J., Serrano L. (2015). Surface-modified nano-cellulose as reinforcement in poly(lactic acid) to conform new composites. Ind. Crop. Prod..

[66] Yu H.-Y., Zhang H., Song M.-L., Zhou Y., Yao J., Ni Q.-Q. (2017). From Cellulose Nanospheres, Nanorods to Nanofibers: Various Aspect Ratio Induced Nucleation/Reinforcing Effects on Polylactic Acid for Robust-Barrier Food Packaging. ACS Appl. Mater. Interfaces.

[67] Jonoobi M., Harun J., Mathew A.P., Oksman K. (2010). Mechanical properties of cellulose nanofiber (CNF) reinforced polylactic acid (PLA) prepared by twin screw extrusion. Compos. Sci. Technol..

[68] Iwatake A., Nogi M., Yano H. (2008). Cellulose nanofiber-reinforced polylactic acid. Compos. Sci. Technol..

[69] Haque M.M.-U., Puglia D., Fortunati E., Pracella M. (2017). Effect of reactive functionalization on properties and degradability of poly(lactic acid)/poly(vinyl acetate) nanocomposites with cellulose nanocrystals. React. Funct. Polym..

[70] John M.J., Anandjiwala R., Oksman K., Mathew A.P. (2013). Melt-spun polylactic acid fibers: Effect of cellulose nanowhiskers on processing and properties. J. Appl. Polym. Sci..

[71] Meng X., Bocharova V., Tekinalp H., Cheng S., Kisliuk A., Sokolov A.P., Kunc V., Peter W.H., Ozcan S. (2018). Toughening of nanocelluose/PLA composites via bio-epoxy interaction: Mechanistic study. Mater. Des..

[72] Khakalo A., Filpponen I., Rojas O.J. (2018). Protein-mediated interfacial adhesion in composites of cellulose nanofibrils and polylactide: Enhanced toughness towards material development. Compos. Sci. Technol..

[73] Nakagaito A.N., Fujimura A., Sakai T., Hama Y., Yano H. (2009). Production of microfibrillated cellulose (MFC)-reinforced polylactic acid (PLA) nanocomposites from sheets obtained by a papermaking-like process. Compos. Sci. Technol..

[74] Nakagaito A., Kanzawa S., Takagi H. (2018). Polylactic Acid Reinforced with Mixed Cellulose and Chitin Nanofibers—Effect of Mixture Ratio on the Mechanical Properties of Composites. J. Compos. Sci..

[75] Robles E., Kánnár A., Labidi J., Csóka L. (2018). Assessment of physical properties of self-bonded composites made of cellulose nanofibrils and poly (lactic acid) microfibrils. Cellulose.

[76] Sethi J., Farooq M., Sain S., Sain M., Sirviö J.A., Illikainen M., Oksman K. (2018). Water resistant nanopapers prepared by lactic acid modified cellulose nanofibers. Cellulose.

[77] Lee K.-Y., Blaker J.J., Bismarck A. (2009). Surface functionalisation of bacterial cellulose as the route to produce green polylactide nanocomposites with improved properties. Compos. Sci. Technol..

[78] Hong J., Kim D.S. (2013). Preparation and physical properties of polylactide/cellulose nanowhisker/nanoclay composites. Polym. Compos..

[79] Raquez J.M., Murena Y., Goffin A.L., Habibi Y., Ruelle B., DeBuyl F., Dubois P. (2012). Surface-modification of cellulose nanowhiskers and their use as nanoreinforcers into polylactide: A sustainably-integrated approach. Compos. Sci. Technol..

[80] Kiziltas A., Nazari B., Erbas Kiziltas E., Gardner D.J., Han Y., Rushing T.S. (2016). Method to reinforce polylactic acid with cellulose nanofibers via a polyhydroxybutyrate carrier system. Carbohydr. Polym..

[81] Bitinis N., Verdejo R., Bras J., Fortunati E., Kenny J.M., Torre L., López-Manchado M.A. (2013). Poly(lactic acid)/natural rubber/cellulose nanocrystal bionanocomposites Part I. Processing and morphology. Carbohydr. Polym..

[82] Herrera N., Salaberria A.M., Mathew A.P., Oksman K. (2016). Plasticized polylactic acid nanocomposite films with cellulose and chitin nanocrystals prepared using extrusion and compression molding with two cooling rates: Effects on mechanical, thermal and optical properties. Compos. Part A.

[83] Aouat T., Kaci M., Devaux E., Campagne C., Cayla A., Dumazert L., Lopez-Cuesta J.M. (2018). Morphological, Mechanical, and Thermal Characterization of Poly (Lactic Acid)/Cellulose Multifilament Fibers Prepared by Melt Spinning. Adv. Polym. Technol..

[84] Hossain K.M.Z., Hasan M.S., Boyd D., Rudd C.D., Ahmed I., Thielemans W. (2014). Effect of cellulose nanowhiskers on surface morphology, mechanical properties, and cell adhesion of melt-drawn polylactic acid fibers. Biomacromolecules.

[85] Kariz M., Sernek M., Obućina M., Kuzman M.K. (2018). Effect of wood content in FDM filament on properties of 3D printed parts. Mater. Today Commun..

[86] Wang Z., Xu J., Lu Y., Hu L., Fan Y., Ma J., Zhou X. (2017). Preparation of 3D printable micro/nanocellulose-polylactic acid (MNC/PLA) composite wire rods with high MNC constitution. Ind. Crop. Prod..

[87] Suryanegara L., Okumura H., Nakagaito A.N., Yano H. (2011). The synergetic effect of phenylphosphonic acid zinc and microfibrillated cellulose on the injection molding cycle time of PLA composites. Cellulose.

[88] Singh A.A., Geng S., Herrera N., Oksman K. (2018). Aligned plasticized polylactic acid cellulose nanocomposite tapes: Effect of drawing conditions. Compos. Part A.

[89] Singh A.A., Wei J., Herrera N., Geng S., Oksman K. (2018). Synergistic effect of chitin nanocrystals and orientations induced by solid-state drawing on PLA-based nanocomposite tapes. Compos. Sci. Technol..

[90] Sung S.H., Chang Y., Han J. (2017). Development of polylactic acid nanocomposite films reinforced with cellulose nanocrystals derived from coffee silverskin. Carbohydr. Polym..

[91] Dhar P., Tarafder D., Kumar A., Katiyar V. (2016). Thermally recyclable polylactic acid/cellulose nanocrystal films through reactive extrusion process. Polymer.

[92] Herrera N., Mathew A.P., Oksman K. (2015). Plasticized polylactic acid/cellulose nanocomposites prepared using melt-extrusion and liquid feeding: Mechanical, thermal and optical properties. Compos. Sci. Technol..

[93] Hervy M., Blaker J.J., Braz A.L., Lee K.-Y. (2018). Mechanical response of multi-layer bacterial cellulose nanopaper reinforced polylactide laminated composites. Compos. Part A.

[94] Abdulkhani A., Hosseinzadeh J., Ashori A., Dadashi S., Takzare Z. (2014). Preparation and characterization of modified cellulose nanofibers reinforced polylactic acid nanocomposite. Polym. Test..

[95] Suryanegara L., Nakagaito A.N., Yano H. (2009). The effect of crystallization of PLA on the thermal and mechanical properties of microfibrillated cellulose-reinforced PLA composites. Compos. Sci. Technol..

[96] Lee K.-Y., Aitomäki Y., Berglund L.A., Oksman K., Bismarck A. (2014). On the use of nanocellulose as reinforcement in polymer matrix composites. Compos. Sci. Technol..

[97] Montrikittiphant T., Tang M., Lee K.Y., Williams C.K., Bismarck A. (2014). Bacterial cellulose nanopaper as reinforcement for polylactide composites: Renewable thermoplastic nanopapreg. Macromol. Rapid Commun..

[98] Hervy M., Bock F., Lee K.-Y. (2018). Thinner and better: (Ultra-)low grammage bacterial cellulose nanopaper-reinforced polylactide composite laminates. Compos. Sci. Technol..

[99] Dhar P., Bhasney S.M., Kumar A., Katiyar V. (2016). Acid functionalized cellulose nanocrystals and its effect on mechanical, thermal, crystallization and surfaces properties of poly (lactic acid) bionanocomposites films: A comprehensive study. Polymer.

[100] Monika, Dhar P., Katiyar V. (2017). Thermal degradation kinetics of polylactic acid/acid fabricated cellulose nanocrystal based bionanocomposites. Int. J. Biol. Macromol..

